# Development and Validation of Markers for the Fertility Restorer Gene *Rf1* in Sunflower

**DOI:** 10.3390/ijms20061260

**Published:** 2019-03-13

**Authors:** Renate Horn, Aleksandra Radanovic, Lena Fuhrmann, Yves Sprycha, Sonia Hamrit, Milan Jockovic, Dragana Miladinovic, Constantin Jansen

**Affiliations:** 1 Department of Plant Genetics, Institute of Biological Sciences, University of Rostock, Albert-Einstein-Str. 3, D-18059 Rostock, Germany; lena.fuhrmann@uni-rostock.de (L.F.); yves.sprycha@uni-rostock.de (Y.S.); 2 Industrial Crops Department, Institute of Field and Vegetable Crops, Maksima Gorkog 30, 21000 Novi Sad, Serbia; aleksandra.dimitrijevic@ifvcns.ns.ac.rs (A.R.); milan.jockovic@ifvcns.ns.ac.rs (M.J.); dragana.miladinovic@ifvcns.ns.ac.rs (D.M.); 3 Strube Research GmbH & Co. KG, Hauptstr. 1, D-38387 Söllingen, Germany; s.hamrit@strube-research.net (S.H.); c.jansen@strube-research.net (C.J.)

**Keywords:** amplicon targeted sequencing, breeding, candidate genes, fertility restoration, marker assisted selection, Polymerase chain reaction Amplification of Multiple Specific Alleles (PAMSA), pentatricopeptide repeat, restorer gene *Rf1*, Sequence Characterized Amplified Region (SCAR), sunflower (*Helianthus annuus*)

## Abstract

Hybrid breeding in sunflowers based on CMS PET1 requires development of restorer lines carrying, in most cases, the restorer gene *Rf1*. Markers for marker-assisted selection have been developed, but there is still need for closer, more versatile, and co-dominant markers linked to *Rf1*. Homology searches against the reference sunflower genome using sequences of cloned markers, as well as Bacterial Artificial Chromosome (BAC)-end sequences of clones hybridizing to them, allowed the identification of two genomic regions of 30 and 3.9 Mb, respectively, as possible physical locations of the restorer gene *Rf1* on linkage group 13. Nine potential candidate genes, encoding six pentatricopeptide repeat proteins, one tetratricopeptide-like helical domain, a probable aldehyde dehydrogenase 22A1, and a probable poly(A) polymerase 3 (PAPS3), were identified in these two genomic regions. Amplicon targeted next generation sequencing of these nine candidate genes for *Rf1* was performed in an association panel consisting of 27 maintainer and 32 restorer lines and revealed the presence of 210 Single Nucleotide Polymorphisms (SNPs) and 67 Insertions/Deletions (INDELs). Association studies showed significant associations of 10 SNPs with fertility restoration (*p*-value < 10^−4^), narrowing *Rf1* down to three candidate genes. Three new markers, one co-dominant marker 67N04_P and two dominant markers, PPR621.5R for restorer, and PPR621.5M for maintainer lines were developed and verified in the association panel of 59 sunflower lines. The versatility of the three newly developed markers, as well as of three existing markers for the restorer gene *Rf1* (HRG01 and HRG02, Cleaved Amplified Polymorphic Sequence (CAPS)-marker H13), was analyzed in a large association panel consisting of 557 accessions.

## 1. Introduction

In the last 30 years, progress in sunflower hybrid breeding has been accompanied by the development of a large number of molecular markers for disease resistance, quality traits, herbicide resistance, and fertility restoration [1]. Sunflower hybrid production exploits heterotic phenomenon (phenotypic superiority of the progeny over its parents). Hybrid development relies on the use of sunflower male-sterile lines (A), maintainer lines (B) for the maintenance of A lines, and fertility restorer lines (R). In sunflowers, commercial hybrids are based worldwide on one source of cytoplasmic male sterility (CMS), the so-called CMS PET1 cytoplasm [2]. CMS PET1 emerged in an interspecific cross between *H. petiolaris* and *H. annuus* [3]. This CMS system is based on interaction between cytoplasmic and nuclear genes. Sunflower CMS lines carry a maternally inherited trait causing failure to produce pollen, while the B line represents a fertile version of A line, an isogenic line having a normal cytoplasm. Pollen fertility in hybrids is regained by crossing a CMS line with a fertility restorer line (R), which is a male fertile pollen parent carrying dominant restorer gene in the nucleus [4]. In the majority of cases, the sunflower restorer line will carry the dominant restorer gene *Rf1*, which has been originally introduced into the sunflower breeding pool by the line T66006-2-1-B [5]. Since then, this has been the major source of fertility restoration in sunflower hybrid breeding [6]. A second dominant restorer gene *Rf2*, essential for full fertility restoration in presence of CMS PET1, was tracked down in the cross of T66006-2-1-B with MZ01398, but further investigations demonstrated that this gene was present in most maintainer and restorer lines [7]. Therefore, molecular efforts have concentrated on developing markers tightly linked to the restorer gene *Rf1* [1]. A large number of different marker systems are available for marker-assisted selection (MAS) in plant breeding [8]. Random Amplified Polymorphic DNA (RAPD) and Amplified Fragment Length Polymorphism (AFLP) markers were identified via bulked segregant analyses and linked to the restorer gene *Rf1* [9]. Cloning and sequencing of closely linked markers (OPK13_454 and OPY10_740) allowed the development of two Sequence Tagged Site (STS) markers HRG01 and HRG02 for marker-assisted selection. Recent studies from Markin et al. [10] investigating accessions present at the Vavilov Institute of Plant Genetic Resources showed that HRG01 worked very well in annual species of the genus *Helianthus*, while HRG02 proved to be generally applicable in perennial species. However, as the two STS markers HRG01 and HRG02 are dominant markers, their use in marker-assisted breeding is limited because heterozygous progenies cannot be distinguished from homozygous. Another RAPD-marker was converted into a Cleaved Amplified Polymorphic Sequence (CAPS) marker [11]. The CAPS marker H13 is codominant, but with 7.7 cM too far away from the restorer gene *Rf1* to be of real use in breeding programs. A Target Region Amplification Polymorphism (TRAP) marker closely linked to *Rf1* could also be identified by Yue et al. [12]. However, TRAP markers are too labor-intensive for large breeding programs. This still leaves a high demand for co-dominant markers closely linked to the restorer gene *Rf1* in sunflowers. The closer the marker is linked to the gene of interest, the greater is its value for MAS, as fewer recombinants will be falsely selected by the marker. In the ideal case, the marker is directly developed from the causal gene, in our case the restorer gene *Rf1*. However, approaches of map-based cloning of the restorer gene *Rf1* have so far only identified closely linked markers [9,11], as well as BAC clones, identified by colony hybridizations forming small contigs around the *Rf1* locus [13,14]. However, with the publication of the reference sunflower genome [15], new possibilities to develop markers from potential candidate genes are available. A prerequisite is the exact localization of the restorer gene *Rf1* in the sunflower genome, which is known to be present on linkage group 13 [11]. 

A number of restorer genes have been isolated from different crop species [16]. Most of these restorer genes belong to the class of pentatricopeptide repeat (PPR) proteins. This is a large family of genes that encodes proteins with a degenerated 35 amino acid domain tandemly repeated up to 30 times, which are involved in translation, RNA splicing, RNA editing, and RNA stability in organelles (mitochondria and chloroplasts) [17,18,19]. PPR-type fertility restorer genes have been cloned in petunia [20], for Ogura and Kosena cytoplasm in *Raphanus sativus* [21,22,23], for Boro II CMS in *Oryza sativa* [24], A1 cytoplasm in *Sorghum bicolor* [25], Honglian CMS in rice [26,27], and nap cytoplasmic male sterility in *Brassica napus* [28]. However, other restorer genes, so-called non-PPR genes, also exist. The *Rf2* gene essential for fertility restoration of the T-cytoplasm in maize represents a mitochondrial aldehyde dehydrogenase [29,30]. Others are the *Rf17* gene encoding an acyl-carrier protein synthase for the Chinese wild rice cytoplasm in rice [31], the restorer gene *Rf1* for LEAD-CMS, also in rice, which represents a glycine-rich protein [32], and finally the *Rf1* gene (*bvORF20*) for the Owen cytoplasm in sugar beet, encoding an OMA1 (overlapping activity with m-AAA protease 1)-like protein that acts post-translational [33,34].

In sunflowers, cytoplasmic male sterility in CMS PET1 is characterized by the expression of CMS-specific 16-kDa-protein encoded by *orfH522*, which is co-transcribed with *atp1* [35,36,37]. Fertility restoration leads to a tissue-specific post-transcriptional reduction of the co-transcript of *atp1* and *orfH522* in the anthers, thereby reducing the 16-kDa-protein [38,39]. The RNA instability of the co-transcript that allows fertility restoration might be due to an interaction with a PPR protein [19], but also a poly(A) polymerase (PAPS) might be a potential candidate as polyadenylation-assisted RNA degradation plays a major role in post-transcriptional control of expression in plant mitochondria [40,41].

In this publication, we would first like to address new possibilities for developing markers based on the available whole sunflower genome sequence [15], and secondly the versatility of markers in different lines and their usability for marker-assisted breeding. By localizing the restorer gene *Rf1* in the sunflower reference genome, we were able to screen the two genomic regions for annotated candidate genes. Nine possible candidates, including *PPR* genes, were identified and the genetic diversity was captured in an association panel by amplicon-targeted next generation sequencing (NGS). Single Nucleotide Polymorphisms (SNPs) significantly associated with fertility restoration were identified and the first new markers were developed. 

## 2. Results

### 2.1. Localization of the Restorer Gene Rf1 in the Sunflower Reference Genome

#### 2.1.1. Identification of BAC Clones by Hybridization with Cloned Markers for the Restorer Gene *Rf1*

RAPD-markers (OP-K13_454A, OP-Y10_740A, OP-H13_337A) and AFLP-markers (E41M48_113A, E42M76_125A, E62M52_249A) linked with the restorer gene *Rf1* [9,11], as well as the BAC end of 067N04, were used to derive overgo probes for colony hybridizations against two BAC libraries, one of the restorer line RHA 325 [42], and one of the maintainer line HA383 (CUGI). In total, 28 BAC clones were identified: six BAC clones of RHA 325 and 22 BAC clones of HA 383 (Table 1). The overgo probes for OP-H13_337A and OP-Y10_740A did not give signals with any BAC clones. All identified BAC clones were sequenced with T7 and SP6 to obtain BAC end sequences for the localization of the restorer gene *Rf1* by using BLAST against the sunflower reference genome [15]. In addition, larger BAC-end fragments obtained by *Bam*HI digests of the identified BACs were cloned and sequenced.

#### 2.1.2. Homology Search against the Reference Sunflower Genome

**Figure 1:**
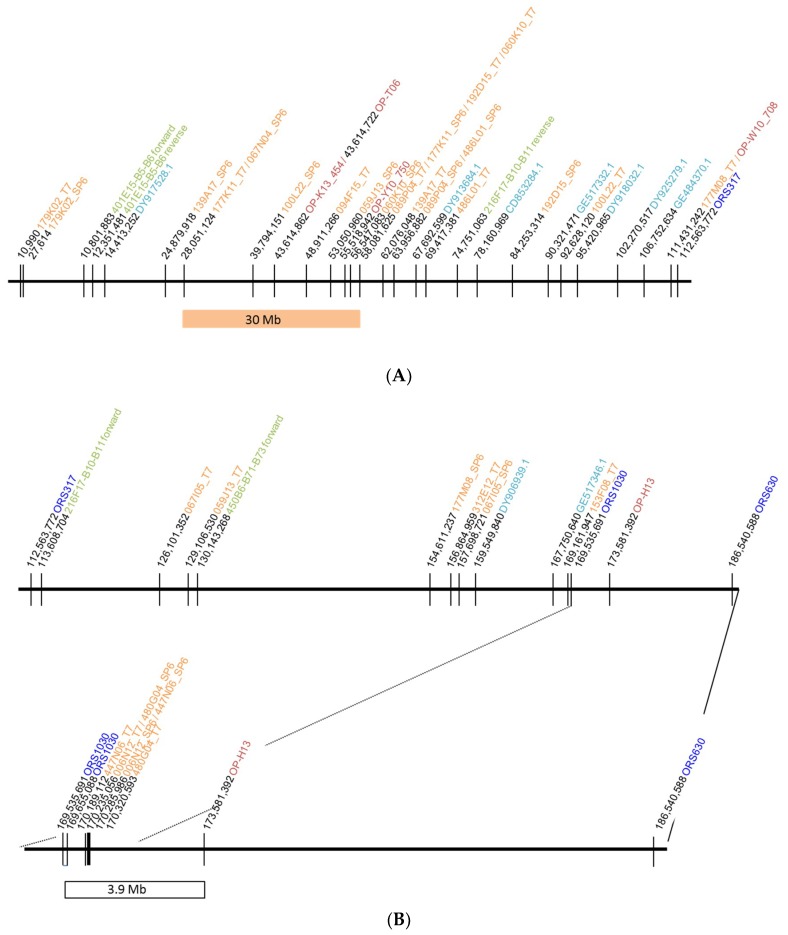
BLAST results of BAC clones and marker sequences against chromosome 13 of the HanXRQ genome sequence. (**A**) region between 10,990 and 112,563,772; (**B**) region between 112,563,772 and 186,540,588. BAC clones (T7 or SP6 sequences) are shown in orange, RAPD markers in red [14], SSR markers in blue [44], and SNP markers in turquoise [43]. The two areas investigated for potential candidate genes for *Rf1* are shown as orange bar and white bar.

Using BAC end sequence data belonging to the identified BAC clones from the BAC libraries of RHA 325 and HA 383 and sequences of markers linked to the restorer gene *Rf1* [9,11], homologies were detected along chromosome 13 of HanXRQ in the genome sequence [15] from position 10,990 to 173,581,392 (Figure 1). Homologies to SNP marker sequences from the SNP array [43] and Simple Sequence Repeat (SSR) markers [44] were included in the graphic for general orientation. The homologies were not as concentrated as expected, which might be because HanXRQ represents a maintainer line and not a restorer line. Recombination events involving larger genome regions may have occurred in the restorer lines. 

However, assuming microsynteny, our major interest focused on two regions of 30 Mb and 3.9 Mb, respectively. These areas showed homology to clusters of BAC clones. In addition, the first region showed homology to markers as OP-K13_454 and OP-Y10_750, which have been successfully used in marker-assisted selection for the restorer gene *Rf1* [9,10], and to the BAC clone 067N04, which had been successfully back mapped to *Rf1*. The second area flanked by ORS1030 and OP-H13 [11] represents a small cluster of BACs with homology to the area. Both markers represent co-dominant markers that had also been directly linked to the restorer gene *Rf1*.

#### 2.1.3. Potential Candidate Genes for *Rf1* in the Annotated Sunflower Reference Genome

The two regions on linkage group 13 of the sunflower genome spanning the areas (1) 28,051,124 (067N04_SP6)–58,081,625 (89P04_T7) and (2) 169,655,088 (ORS1030)–173,581,392 (OP-H13) were selected as potential locations of the restorer gene *Rf1*, and therefore regarded as most promising for future marker developments. Based on the annotation of the HanXRQ genome sequence (https://www.heliagene.org/HanXRQ-SUNRISE/), nine potential candidate genes located within the two genomic regions were selected for next generation sequencing (NGS) by custom targeted amplification (Table 2). These represent six putative pentatricopeptide repeat genes. In addition, a gene for a tetratricopeptide-like helical domain, a probable aldehyde dehydrogenase 22A1, and a probable poly(A) polymerase 3 (PAPS3) were also present in these two areas. The genes, as well as 2,000 bp upstream and 500 bp downstream, were sequenced by NGS in order to obtain information about the coding sequences, as well as about potential regulatory sequences. In total, 58,209 bp covering these genes were selected for sequencing by an NGS approach and analyzed for SNPs and INDELs that can be used for development of markers. 

### 2.2. Association Studies for SNPs Linked to the Restorer Gene Rf1 Performed

#### 2.2.1. SNP Detection in the Sunflower Association Panel

Amplicon-targeted next generation sequencing of nine potential candidate genes for *Rf1* in an association panel consisting of 27 maintainer and 32 restorer lines (Supplementary Table S1) and alignment to the HanXRQ genome sequence identified 277 variants (210 SNPs and 67 INDELs) (Table 3). The haplotype variation among the maintainer and restorer lines based on the SNPs is shown in Supplementary Table S2. The four genes (HanXRQChr13g0392791, HanXRQChr13g0393411, HanXRQChr13g0394161, HanXRQChr13g0394751) in the large region between 28,051,124 bp (067N04_SP6–58,081,625 bp (89P04_T7) were highly conserved from the sequence and showed only 23 variants (4 SNPs and 19 INDELs). These genes encode two pentatricopeptide repeat proteins, PPR791 and PPR161, and the probable aldehyde dehydrogenase 22A1, as well as the probable poly(A) polymerase 3 (PAPS3). In contrast, the five candidate genes (HanXRQChr13g0418841, HanXRQChr13g0418851, HanXRQChr13g0418861, HanXRQChr13g0419621, HanXRQChr13g0419631) in the 3.9-Mb-region between 169,655,088 bp (ORS1030)–173,581,392 bp (OP-H13) proved to be highly polymorphic, with a total of 254 variants (206 SNPs and 48 INDELs). Even the exon regions still showed 76 SNPs and 8 INDELs. These genes encode four pentatricopeptide repeat proteins, PPR841, PPR861, PPR621 (also named PP198), and PPR631. The fifth gene (HanXRQChr13g0418851) is annotated as a gene encoding a tetratricopeptide-like repeat helical domain. However, the corresponding gene in *Arabidopsis* (At1g12700) represents a pentatricopeptide repeat protein.

#### 2.2.2. Populations Structure

**Figure 2:**
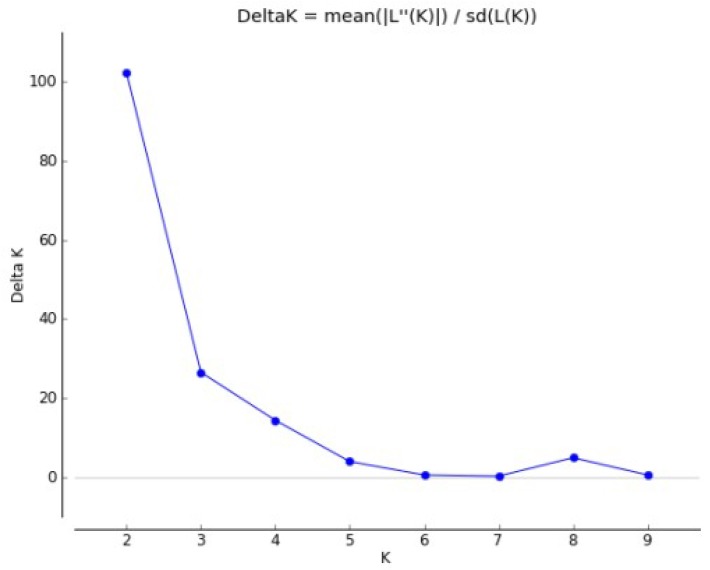
Delta Evanno statistics for number of presumed subpopulations ranging from 1 to 10 in the sunflower association panel.

**Figure 3:**
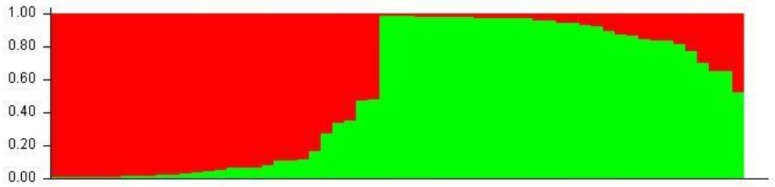
STRUCTURE output for sunflower association panel with K = 2 clusters based on 34 polymorphic SSR primer combinations. Two subpopulations were identified in the sunflower association panel composed of 59 accessions in total. The first subpopulation (sp1) is labeled with red color and the second subpopulation (sb2) with green color.

The population structure of the association panel consisting of 27 maintainer und 32 restorer lines was analyzed based on 34 microsatellite markers using the program STRUCTURE V.2.3.4 to avoid false positive associations due to the population structure. Two microsatellites per chromosome were used to obtain a good coverage of the genome. By use of the Evanno method, two probable subpopulations were identified in the sunflower association panel using STRUCTURE V.2.3.4. (Figure 2 and Figure 3). Twenty-eight sunflower accessions were grouped in the first subpopulation (sp1). The majority of sunflower accessions belonging to this group were restorer lines, except for UGA-SAM1-171 and D-75-10. Thirty-one sunflower accessions were grouped in the second subpopulation (sp2) with the majority being maintainer lines (except for six lines: GN-0778, RHA 282, UGA-SAM1-041, UGA-SAM1-149, UGA-SAM1-181, and UGA-SAM1-278). The mean value of the fixation index (Fst) of the first subpopulation was 0.11, while Fst for the second subpopulation was higher with a value of 0.26.

#### 2.2.3. Association of SNPs with Fertility Restoration

**Figure 4:**
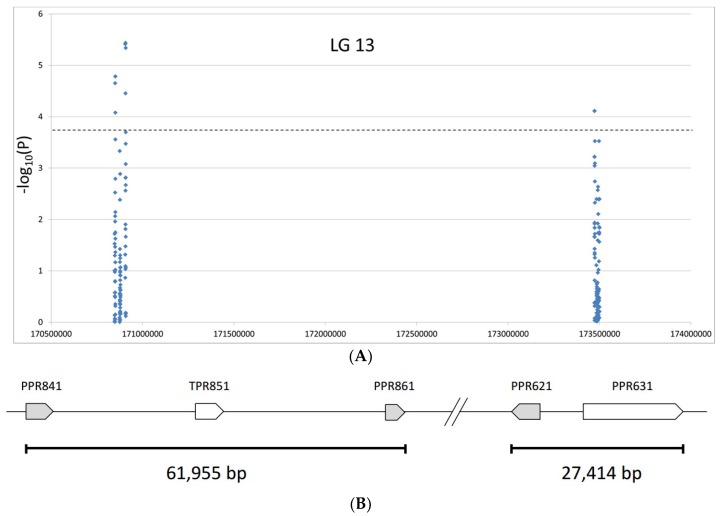
Distribution of SNPs present in five candidate genes for *Rf1* in the 3.9-Mb-region (169,655,088–173,581,392) on linkage group 13. (**A**) Manhattan plot. Dotted line represents the threshold (*p* < 1.81 × 10^−4^) after Bonferroni correction. (**B**) Schematic distribution of the five candidate genes in the 3.9-Mb-region derived from the HanXRQ genome annotation.

Association studies were performed using the general linear model (GLM) in TASSEL V5.0, taking into account the population structure (Q) as calculated by STRUCTURE V.2.3.4. Out of 277 variants detected in the sequences of the nine candidate genes for the restorer gene *Rf1*, 74 SNPs were found to be significantly associated with restoration of fertility at *p* < 0.05. In the 30-Mb-region, only three SNPs at position 56,431,576, 56,432,393, and 56,433,210, all located in *PAPS3*, were found to be associated with male fertility at this significance level. The remaining 71 SNPs were all residing in the 3.9-Mb-region. The Manhattan plot shows the distribution of all SNPs in the 3.9-Mb-region (Figure 4). Twenty-one SNPs still showed associations at *p*-values lower than 10^−3^ (Table 4). After applying the Bonferroni correction, 10 SNPs were finally left above the calculated threshold (*p* < 1.81 × 10^−4^). All of these highly significant associated SNPs were located in three of the pentatricopeptide repeat genes in the 3.9-Mb-region (169,655,088–173,581,392 bp): four each were observed in *PPR861* and *PPR841*, but only two in *PPR621* (Table 4). These 10 SNPs would be the most interesting ones to be used for marker development. Looking at the haplotype of these with the fertility restoration associated SNPs, 85.2% of the maintainer lines in the association panel showed the same haplotype as HanXRQ (Table 5) and 81.3% of the restorer lines showed a different unique haplotype if three of the SNPs are handled in a more relaxed manner.

### 2.3. Development of New SNP-Based Markers for the Restorer Gene Rf1

#### 2.3.1. PAMSA Marker System

**Figure 5:**
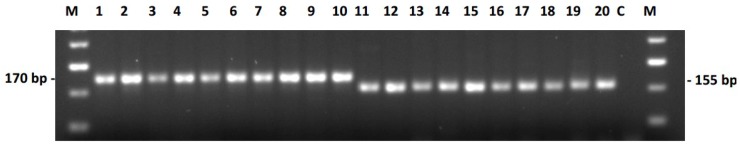
PAMSA marker derived from BAC end sequence of 67N04. 1-10 represent restorer lines, 11-20 maintainer lines; 1 = RHA 273, 2 = RHA 294, 3 = RHA 298, 4 = RHA 311, 5 = RHA 325, 6 = RHA 354, 7 = RHA 363, 8 = RHA 376, 9 = RHA 396, 10 = RHA 858, 11 = HA 228, 12 = HA 249, 13 = HA 285, 14 = HA 288, 15 = HA 307, 16 = HA 319, 17 = HA 320, 18 = HA 425, 19 = HA 442, 20 = HA 446, C = negative control, M = 50 bp marker New England BioLabs^®^.

Polymerase chain reaction (PCR) Amplification of Multiple Specific Alleles (PAMSA) markers represent a SNP-based three primer system, where the addition of a 15-bp-tail at the 5′ end of one of the forward primer allows an allele-specific size differentiation of the PCR products [45]. The PAMSA marker, named 67N04_P, was created for the detection of a G to A mutation in the BAC-end sequence 67N04-B2 *Bam*HI (RHA 325, [13]) observed in comparison to HanXRQ. Detection of this SNP was performed in agarose gels in order to make it accessible even in simply equipped laboratories. The codominant marker created enabled discrimination between the restorer line RHA 325 and the maintainer line HA 342 due to difference the 15-bp-difference in the forward primer length. A band of 170 bp was amplified in the restorer line, while a band of only 155 bp was visible in the maintainer line (Figure 5). 

This marker was later tested in the large association panel (see Section 2.4. Versatility of markers for the restorer gene *Rf1*), in which it amplified a band of 170 bp in lines that contained the nucleotide A (as present in RHA 325) and a band of 155 bp for maintainer carrying the nucleotide G.

#### 2.3.2. New SNP-Based Markers from the Association Analyses

**Figure 6:**
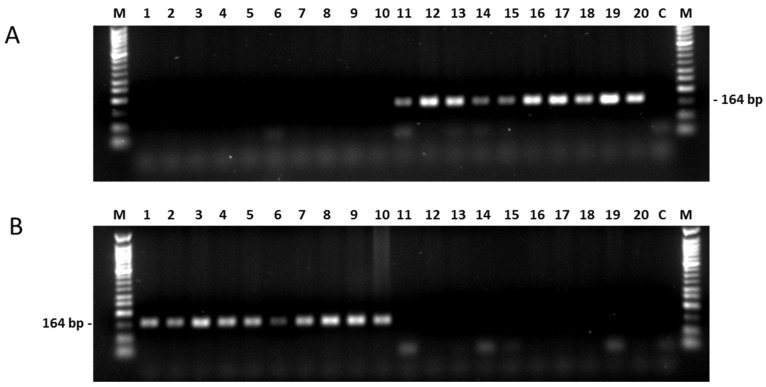
SNP markers PPR621.5M and PPR621.5R derived from *PPR621*. (**A**) Marker PPR621.5M for the maintainer lines. (**B**) Marker PPR621.5R for the restorer lines carrying the *Rf1* gene. 1 = RHA-282, 2 = UGA-SAM1-012, 3 = UGA-SAM1-024, 4 = UGA-SAM1-100, 5 = UGA-SAM1-121, 6 = UGA-SAM1-136, 7 = UGA-SAM1-169, 8 = UGA-SAM1-186, 9 = UGA-SAM1-191, 10 = UGA-SAM1-198, 11 = Arrowhead, 12 = CM104, 13 = CM526, 14 = Krasnodarets, 15 = UGA-SAM1-082, 16 = UGA-SAM1-109, 17 = UGA-SAM1-110, 18 = UGA-SAM1-140, 19 = UGA-SAM1-183, 20 = UGA-SAM1-273, C = negative control, M = 50 bp marker New England BioLabs^®^.

For all 10 SNPs significantly associated with fertility restoration primers were derived and tested in RHA 325 and HA 342 (data not shown), but only for the SNP PPR621.5 reproducible polymerase chain reaction (PCR) products were amplified that differentiated immediately between this restorer and this maintainer line. Two Sequence Characterized Amplified Region (SCAR) markers, PPR621.5M and PPR621.5R, were successfully developed for detection of G for maintainers (PPR621.5M) and C for restorers (PPR621.5R) based on the SNP at position 173,473,513 in the pentatricopeptide repeat candidate gene PPR621 (PP198; HanXRQChr13g0419621). Separate PCR reactions had to be performed for detection of the presence of the G or C nucleotide. In both reactions, a common reverse primer was used for the amplification, while the difference was in the sequence of the allele-specific forward primers. For detection of the G nucleotide using the primer combination PPR621.5_F1 and PPR621.5_Rev (PPR621.5M), a band of approximately 164 bp was amplified in the maintainer line HA 342, while no band was visible in the restorer line RHA 325 (Figure 6). In contrast, primer combination PPR621.5_F2 and PPR621.5_Rev (PPR621.5R) designed for the detection of the C nucleotide at 173,473,513 position of the sunflower genome also amplified a band of 164 bp, but only in the restorer line RHA 325 and not in HA 342. First validation of the markers was performed in the association panel of 59 sunflower accessions, which had been used for the next generation sequencing of the potential *Rf_1_* candidate genes (Figure 6). For the majority of the tested accessions, the results obtained by use of NGS and by use of PCR matched each other. Most of the restorer lines showed a C at position 173,473,513 with the exception of four out of 32 restorer lines. These four restorer lines (GN-0778, UGA-SAM1-181 (RHA 311), UGA-SAM1-199 (R-206), and UGA-SAM1-278 (INRA line SF_295)) possessed the nucleotide G at this position, as verified by use of both methods, NGS and PCR.

However, also in very few cases inconsistencies were observed between the NGS and the PCR results. For the two maintainer lines UGA-SAM1-219 (HA1) and UGA-SAM1-224 (HA 248), a band was amplified by use of PPR621.5_F1 and PPR621.5_Rev, which was designed for detection of the G nucleotide as expected in the reference maintainer genotype and other maintainer accessions, but NGS results had shown the nucleotide C for these two lines. Similarly, the two methods did not give matching results for the two restorer lines UGA-SAM1-207 (RHA 419) and UGA-SAM1-242 (RHA 355), where the presence of the nucleotide G was established by NGS, and the nucleotide C by PCR. As only four lines showed inconsistencies between NGS and PCR, we observed 93% accordance between the two methods.

### 2.4. Versatility of Markers for the Restorer Gene Rf1

#### Testing Markers in A Large Association Panel for Their Efficiency

A large sunflower association panel containing 557 diverse accessions was used for validation of molecular markers created for the detection of the *Rf1* gene (Supplementary Table S3). Within the panel were maintainer sunflower lines with no *Rf* genes and restorer sunflower lines that harbored different *Rf* genes. However, bearing in mind that *Rf1* is widely used in modern sunflower hybrid production (Yue et al. 2010), it is expected that most restorer lines possess the *Rf1* gene. Molecular markers used for validation were: STS-markers HRG01 (derived from RAPD fragment OPK13_454), HRG02 (converted from RAPD fragment OPY10_740) [9], OPH13_337 (CAPS marker H13 *Hinf*I developed from OPH13 337) [11], orfH522-CMS for CMS PET1, in comparison to the newly described co-dominant PAMSA marker 67N04_P, as well as the SCAR-markers PPR621.5R and PPR621.5M. The banding pattern of the restorer line RHA 325 (UGA-SAM1-114) was used as reference for restorer lines carrying the *Rf1* gene. For the maintainer profiles, HA 342 served as reference. The restorer line RHA 280 (UGA-SAM1-008) harboring the *Rf3* gene for all markers showed a maintainer profile. 

**Figure 7:**
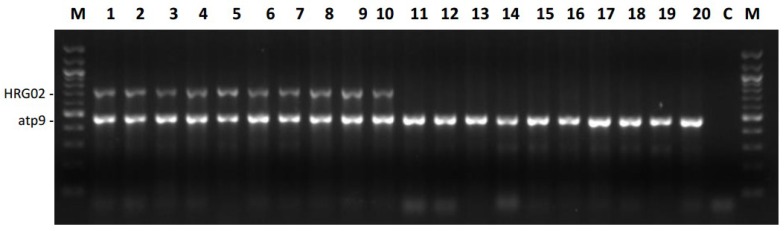
Marker HRG02 for the fertility restorer gene *Rf1.* Marker HRG02 (derived from Y10) for the restorer *Rf1* was analyzed combined with primers for *atp9* used as internal control. 1 = RHA 273, 2 = RHA 294, 3 = RHA 298, 4 = RHA 311, 5 = RHA 325, 6 = RHA 354, 7 = RHA 363, 8 = RHA 376, 9 = RHA 396, 10 = RHA 858, 11 = HA 228, 12 = HA 249, 13 = HA 285, 14 = HA 288, 15 = HA 307, 16 = HA 319, 17 = HA 320, 18 = HA 425, 19 = HA 442, 20 = HA 446, C = negative control, M = 100 bp marker New England BioLabs^®^.

Markers 67N04_P and HRG02 provided the most similar results concerning the marker-trait association (Supplementary Table S3). PAMSA marker 67N04_P amplified a band of 170 bp in 130 accessions (including heterozygous accessions), identifying them as potential restorer lines, while HRG02 amplified a band of approximately 738 bp in 127 sunflower lines (Table 6, Figure 7). For these two markers, profiles obtained matched in 98.4 % of all accessions. HRG01, also previously linked to *Rf_1_* in RHA 325 [9], amplified a band of 462 bp in 100 sunflower lines of the association panel (Table 6, Supplementary Table S3). CAPS-marker H13 *Hinf*I proved to be the most inefficient marker for discrimination between restorer and maintainer accessions.

## 3. Discussion

Marker-assisted assessment of breeding pools includes genotyping of cultivars, determination of purity, and estimation of genetic diversity for selecting parents in breeding programs [46]. Hybrid breeding is most efficient if separate breeding pools for restorer and maintainer lines can be established to maximize use of heterosis (hybrid vigor) [47].

In this study, we focused on the development of new, especially codominant, markers linked to the fertility restorer gene *Rf1* in sunflowers, which are needed to develop lines for the restorer pool. To use the information from the sunflower reference genome [15], it was first necessary to locate *Rf1* in the genome by sequence homology to known markers and BAC clones linked to the restorer gene [14]. Two regions, (1) 28,051,124 (067N04_SP6)–58,081,625 (89P04_T7) and (2) 169,655,088 (ORS1030)–173,581,392 (OP-H13), were identified as possible locations of the restorer gene *Rf1* in the sunflower genome. This first seemed unusual as the regions are 114 Mb apart. However, the homology searches were performed against the sunflower reference genome of the maintainer line HanXRQ. Major recombination events involving large genomic regions may have occurred in the introduction of the restorer gene *Rf1* that could perhaps explain two separate regions with homologies. However, false assemblies of contigs on chromosome 13 during the whole genome assembly cannot be excluded either. Whole genome sequencing of additional maintainer and restorer lines and comparison to the reference maintainer genome [15] are necessary and could reveal this. However, another fact might also point in this direction. Huge efforts have been undertaken to develop codominant markers for the *Rf1* gene, but only the CAPS marker H13, which maps more than 7 cM away, could be developed. This could be also explained by larger rearrangements around the restorer gene locus. 

Association mapping has proven to be a very useful tool in plant breeding to identify marker-trait associations [48]. In sunflowers, association studies have been successfully performed genome-wide for domestication traits [49], as well as based on candidate genes for branching [50], for flowering time [51,52], and for resistance against *Sclerotinia sclerotiorum* [53]. Here, we report about the first association study based on nine candidate genes for fertility restoration in sunflowers. In the two identified locations in the genome for *Rf1*, the 30-Mb-region and the 3.9-Mb-region, nine potential candidate genes could be identified. Six of them belonged to the class of pentatricopeptide repeat genes, which are involved in post-transcriptional processes in mitochondria and chloroplasts [18]. A number of restorer genes isolated from different species belong to this large family of *PPR* genes [16]. The cloned restorer genes are members of a subgroup within the P-type *PPR* gene family named *Restorer of Fertility-Like* (*RFL*) [54], which show “nomadic” characteristics and diversifying selection that allows these *PPR* genes to adapt fast to interact with newly occurring CMS-specific proteins [19,55]. In contrast, other *PPR* gene families (non-Rf) tend to be conserved [56]. Three other potential candidates were located in the 30-Mb-region and 3.9-Mb-region: a tetratricopeptide-like repeat helical domain gene, which is closely related to the *PPR* genes, a probable aldehyde dehydrogenase 22A1 (ALDH22A1), and a probable poly(A) polymerase 3 (PAPS3). Aldehyde dehydrogenases play a role in detoxification of aldehydes. In maize, the *Rf2* gene restoring pollen fertility in the presence of the T-cytoplasm proved to be ALDH2B2 [29,30]. ALDH 22A1 (AT3G66658) has been well-characterized in *Arabidopsis thaliana*, where it showed a cytoplasmic localization and is apart from other tissues, also expressed in anthers [57]. The poly(A) polymerase 3 (PAPS3) also represents a very interesting candidate gene, as polyadenylation at the 3′ end of RNAs influences the stability of transcripts. Of the four poly(A) polymerases described in *Arabidopsis thaliana*, PAPS3 (AT3G06560) represents a cytoplasmic PAPS, which lacks the C-terminus containing the putative nuclear localization domain [58]. PAPS3 is expressed in pollen, implicating an important role in development of male gametes. 

Our association studies including nine candidate genes for *Rf1* demonstrated that the three pentatricopeptide repeat genes *PPR621* (HanXRQChr13g0419621, PP198), *PPR841* (HanXRQChr13g0418841), and *PPR861* (HanXRQChr13g0418861), all located in the 3.9-Mb-region (169,655,088–173,581,392), are the most promising candidate genes for the restorer gene *Rf1* in sunflowers. These three genes showed the highest numbers of SNPs significantly associated with fertility restoration (*p* < 1.81 × 10^−4^). By looking for annotated *PPR* genes in high Fst–regions of whole sunflower genome sequence data, Owens et al. [59] also narrowed down the area of interest for the restorer gene *Rf1* to 10 pentatricopeptide repeat genes. However, due the high stringency, no SNPs were available for the final tagging of the right gene. These 10 candidate genes included four genes (HanXRQChr13g0419621, HanXRQChr13g0419631, HanXRQChr13g0418841, HanXRQChr13g0418861) that we looked at, as well, supporting our choice of candidate genes. We used 120x genome coverage in the amplicon-targeted next generation sequencing, giving us very solid data for our calculation regarding the association of SNPs with fertility restoration. By this we were able to further reduce the number of candidate genes for *Rf1* to three (*PPR621*-HanXRQChr13g0419621, *PPR841*-HanXRQChr13g0418841, and *PPR861*-HanXRQChr13g0418861). However, due to the high homologies to restorer sequences in the 30-Mb-region we cannot exclude that in restorer lines additional major recombination events in comparison to HanXRQ might have occurred, introducing other relevant genes near or into this 3.9-Mb-area.

Testing all 10 with fertility restoration associated SNPs, one of the SNPs at position 173,473,513 in *PPR621* (HanXRQChr13g0419621) was successfully used to develop two SCAR markers (PPR621.5R and PPR621.5M) that allowed the identification of restorer and maintainer lines in separate PCR reactions. In the near future, this SNP can also be used to develop a codominant PAMSA marker or a Kompetitive Allele Specific Polymorphic (KASP) marker [60,61], which would be very interesting for studies on seed purity. Future work will also show the usefulness of the other nine SNPs associated with fertility restoration for marker development. A recent comparison of the three SNP-based marker systems, namely TaqMan, KASP, and rhAmp, might be helpful in selecting the most efficient marker system regarding cost, sensitivity, and reliability [62].

Versatility of newly developed markers in other restorer lines was the next interesting point to be addressed for plant breeding. All markers for the restorer gene *Rf1* in sunflower had so far been developed on the base of segregation in a biparental population [9,11]. Differences that allowed the marker development could have been specific to the cross used. Therefore, it was necessary to test the versatility of these markers in a larger association panel. The newly developed SCAR markers PPR621.5R and PPR621.5M in this study were based on their occurrence in a broader association panel of 59 accessions and should already be more reliable. However, to test the versatility of the markers, we applied them in a large diverse association panel of 557 accessions. Most, but not all lines carrying CMS PET1, showed bands with the STS markers HRG01 and HRG02 for *Rf1*. One reason for this might be the presence of the fertility restorer gene *Rf3* in the association panel, which had been first described in the confectionary line RHA 280 [63]. *Rf3* also allows full fertility restoration in the presence of CMS PET1. Markers linked to *Rf3*, located on linkage group 7, have been identified [64] and could be used to verify this. In addition, *Rf5*, a new restorer gene also located on LG13, has been detected to be not allelic to *Rf1* [65] and could be alternatively present in these lines. In addition, this might also be an explanation for the four restorer accessions (GN-0778, UGA-SAM1-181, UGA-SAM1-199, and UGA-SAM1-278) that did not have the expected C nucleotide at position 173,473,513.

The implementation of markers into breeding programs has been a challenge since the beginning, as researchers and plant breeders have divergent demands and laboratory equipment [46]. In our study, the three newly developed markers PAMSA 67N04_P, PPR621.5R, and PPR621.5M proved to be very valuable tools for marker-assisted selection, because PAMSA 67N04_P represents a co-dominant marker and PPR621.5R and PPR621.5M work in combination, even though they required separate PCR reactions, also allow the identification of heterozygous plants. Hopefully these markers will find their entrance into sunflower hybrid breeding programs as they are easy and reliable to handle in a large variety of sunflower breeding materials.

## 4. Material and Methods

### 4.1. Plant Material for Association Mapping and Validation

The small association panel of 59 accessions for the NGS approach consisted of 45 accessions that are part of the UGA-SAM1 population (Supplementary Table S1). These accessions were obtained from the Germplasm Resources Information Network (GRIN; https://www.ars-grin.gov/) of the USDA National Plant Germplasm System (NPGS), described in Seiler et al. [66]. An additional 14 accessions came from the Plant Gene Resources of Canada (Germplasm Resources Information Network–Canadian version GRIN-CA) (http://pgrc3.agr.gc.ca/index_e.html). The larger sunflower association panel of 557 accessions (Supplementary Table S3) was generated from the same two genetic resources. Field trials were performed near Schlanstedt, Germany, by the company Strube GmbH. Leaves were harvested and stored at −20 °C for DNA extraction. Genomic DNA of the 557 sunflower accessions was isolated according to Doyle and Doyle [67]. Concentration of the isolated DNA was adjusted to 100 ng/µL and was used for amplification with different markers (Supplementary Table S3). 

STS markers, HRG01 and HRG02, were amplified as described by Horn et al. [9] for validation in the large association panel. HRG01 marker was used in a duplex PCR with an internal control *coxII* and annealing temperature set to 60 °C. HRG02 marker was used in a duplex PCR with an internal control *atp9* and annealing temperature set to 65 °C. CAPS marker H13 and *orfH522* were used as described by Kusterer et al. [11] and Reddemann and Horn [68], respectively. Amplification products were separated on ethidium bromide stained 2% agarose gel (Agarose NEEO ultra-quality Roth^®^, Karlsruhe, Germany). 

### 4.2. Cloning and Sequencing of Markers Linked to the Restorer Gene Rf1

RAPD- and AFLP-markers were cloned and sequenced as described by Horn et al. [9]. Sequences were obtained by Sanger sequencing using T7 and SP6 primers. The sequences were used to develop overgo probes (Supplementary Table S4) that could be radioactively labelled with ^32^P-dATP and ^32^P-dCTP to be used as probes for colony hybridization against two BAC libraries (https://www.ncbi.nlm.nih.gov/probe/docs/techovergo/, [14]). One BAC library had been prepared from high molecular weight DNA of the restorer line RHA 325 [42,69]. The second BAC library for the maintainer line HA 383 was obtained from the Clemson University Genomic Institute (CUGI), SC, USA. BAC ends of the positively identified BAC clones were sequenced using T7 and SP6 by CUGI. Larger BAC-ends were cloned by *Bam*HI digests of the original *HindIII* BAC clones and ligation into the pUC18 vector [13]. These BAC-ends were also Sanger sequenced using SP6, but also applying additional internal primers [14].

### 4.3. Microsatellite Markers

The association panel of 59 accessions was analyzed by 34 SSR loci (Supplementary Table S5). The quality criteria for the SSR markers, also called microsatellite markers, were good amplification products, amplification of one locus, and high polymorphism within the association panel. Most of the microsatellites had been used before in association studies in sunflowers [70,71]. Additional ones were obtained from the publication of the sunflower reference map [44] in order to have two microsatellite markers for each of the 17 chromosomes in sunflower. SSRs were amplified by PCR and separated using the DNA Analyzer 4300 (LI-COR Biosciences, Lincoln, NE, USA), as described in Sajer et al. [72].

### 4.4. PAMSA Marker

Polymerase chain reaction (PCR) Amplification of Multiple Specific Alleles (PAMSA) [44] markers was developed based on a BAC (Bacterial Artificial Chromosome)-end sequence 67N04-B2 *Bam*HI (RHA325) [13,14]. Briefly, three unlabeled primers were designed: two forward and a common reverse primer (Supplementary Table S5). Forward allele-specific primers contained an additional destabilizing mismatch within the first five bases of the 3′ end. A mismatch was made by following pattern: A and T → C, G → A, and C → T [73]. In addition, one of the forward primers contained a tail at the 5′ end. This was done to enable differentiation between the two alleles based on difference in length of the amplification product. Primers were designed using Primer3 (http://bioinfo.ut.ee/primer3-0.4.0/). PCR mixture of 15 µL was used for amplification with PAMSA marker. The mixture contained 2x PCR buffer, 0.2 µM dNTPs, 0.4 µM of each primer, 2.4 U of Taq DNA polymerase (New England BioLabs^®^, Ipswich, MA, USA and 100 ng of genomic DNA. Amplification was performed in Applied Biosystems GeneAmp^®^ PCR System 2700 Thermal Cycler under following conditions: 3 min denaturation at 94 °С, followed by 40 cycles of 1 min denaturation at 94 °С, 1 min annealing at 65 °С, 30 s polymerization at 72 °С; followed by a 7 min period of elongation at 72 °С. The amplification products were separated on ethidium bromide stained 3% agarose gel (Agarose NEEO ultra-quality Roth^®^, Karlsruhe, Germany) at 120 V for 60 min.

### 4.5. SCAR Markers

SNP-based markers were developed for the detection of the SNP G to C at position 173,473,513 of the sunflower genome sequence (https://www.heliagene.org/HanXRQ-SUNRISE/, [15]). This SNP was found in the sequence of the PPR621, a pentatricopeptide repeat candidate gene. Created SNP-based markers, PPR621.5M and PPR621.5R, consist of allele-specific primers, two forward and one common reverse primer (Supplementary Table S6). In addition, a second SNP G to A was present in the forward primer sequence, which was detected at the position 173,473,493 of the sunflower genome (Supplementary Table S2). Primers were designed by use of program Primer3 (http://bioinfo.ut.ee/primer3-0.4.0/). PCR mixture (15 µL) used for amplification was the same as with the PAMSA marker (except that in this reaction only two primers were added to the mixture). Amplification was performed in Applied Biosystems GeneAmp^®^ PCR System 2700 Thermal Cycler under the following conditions: 2 min denaturation at 94 °С, followed by 30 cycles; 30 sec denaturation at 94 °С, 30 sec annealing at 62 °С, 1 min polymerization at 72 °С; followed by a final 4 min period of elongation at 72 °С. The amplification products were separated on ethidium bromide stained 2% agarose gels (Agarose NEEO ultra-quality Roth^®^, Karlsruhe, Germany) at 120 V for 60 min.

### 4.6. Population Structure

Population structure in the sunflower association panel of 59 accessions was analyzed based on microsatellite data (34 SSR primer combinations) by use of the admixture model implemented in STRUCTURE V.2.3.4. [74]. Ten independent replications were performed for the analysis of the mean k-value (an assumed fixed number of subpopulations) from 1 to 10. STRUCTURE was set to 50,000 as burn-in time, followed by 100,000 Markov Chain Monte Carlo (MCMC) iterations [51]. The optimal number of subpopulations was determined by use of STRUCTURE HARVESTER [75] applying the Evanno method [76]. Alignment of replicates obtained as the result of the STRUCTURE analysis for a determined number of assumed subpopulations was performed in CLUMPP [77].

### 4.7. Amplicon Sequencing

DNA extraction, amplicon targeted sequencing, and SNP/INDEL analyses were performed as contract work by LGC Genomics, Berlin, Germany. DNA from sunflower leaves of 59 accessions (Supplemental Table S1) was extracted according to a house-intern protocol. Amplicon-targeted next generation sequencing, including primer designs and library preparations for nine potential candidate genes for the restorer gene *Rf1* (Table 2), was performed using the Ovation Custom Target Enrichment System (NuGen Technologies, Tecan Group Ltd., Männedorf, Switzerland). Sequencing was done on a MiSeq V3 subunit (2 × 300 bp) with 120× coverage. Afterwards, all library groups were first demultiplexed using Illumina bcl2fastq 2.17.1.14 software. Here, 1 or 2 mismatches or Ns were allowed in the barcode read when the distances between the barcodes allowed for it. Adapter remnants were clipped from all reads. Read lengths < 100 bases were discarded. A quality trimming of the adapter clipped Illumina reads followed. This included trimming of reads at the 3′-end to get a minimum average Phred quality score > 20 over a window of 10 bases. Reads with final length < 20 bases were discarded. Quality reports were generated for all FASTQ files by the FastQC software. In addition, a read_counts.xlsx file containing all read counts for all samples was generated. Read counts for the SNPs in the nine genes of the 59 genotypes by NGS are shown in Supplementary Table S7.

### 4.8. SNP/INDEL Detection

SNP/INDEL detection was done by LGC Genomcis. Alignment of quality trimmed reads was performed against the genome reference sequence using Burros-Wheeler Aligner BWA-MEM version 0.7.12 (http://bio-wa.sourceforge.net). BAM formatted alignment files were generated by BWA. *Helianthus annuus* XRQ genome assembly v1r1 (https://www.heliagene.org/HanXRQ-SUNRISE/downloads/1.2/ HanXRQr1.0-20151230-EGN-r1.2.zip) served as reference. Variant discovery and genotyping of samples were achieved by the use of the software Freebayes v1.0.2-16 (https://github.com/ekg/freebayes#readme). Spreadsheets and VCF files containing the variant calls were generated.

### 4.9. Association Studies

Marker-trait associations were made by jointly analyzing genotypic and phenotypic data. Association studies were performed with the software package TASSEL V5.0 [78] by use of the General Linear Model (GLM). Minor allele frequency (MAF) > 0.05 was used to filter SNPs prior to analysis, while Q was extracted from the results of the previous population structure analysis (CLUMPP). Bonferroni correction was calculated by dividing 0.05 with the number of total SNPs detected. It was applied for defining a significance threshold of *p* ≤ 1.81 × 10^−4^ for association between detected SNPs and fertility restoration. The Manhattan plot was generated in Excel based on p-values obtained from TASSEL. For the Manhattan plot, the negative logarithm of the *p*-values [−log_10_(P)] from the association studies were plotted against the genomic locations of the SNPs.

## 5. Conclusions

The publication of the reference sunflower genome [15] represents a milestone in sunflower breeding, as it has opened up new and fast possibilities to develop markers for breeding purposes, which we here demonstrated for the development of markers for the restorer gene *Rf1*. After identification of two potential regions for the localization of the restorer gene *Rf1*, potential candidate genes could be addressed in the annotated genome and used for amplicon-targeted next generation sequencing. Association studies using the identified SNPs detected significant associations with three *PPR* genes (HanXRQChr13g0419621, HanXRQChr13g0418841, and HanXRQChr13g0418861). One of these might encode the *Rf1* gene. The SNPs now represent a valuable tool for the development of further markers linked to the restorer gene *Rf1*, which we proved to be possible by successfully designing and testing the new SCAR-markers PPR621.5R and PPR621.5M, specific for restorer or maintainer lines, respectively. 

For a broad application of markers linked to the restorer gene *Rf1*, it is necessary that these markers are not restricted to the biparental population they have been developed in, but are applicable in a wide spectrum of lines. Markers developed based on association, per se, will have a broader possibility of applications. We here successfully tested formerly published markers linked to the restorer gene *Rf1*, as well as three newly developed markers in a diverse association panel of 557 accessions, and could observe wide versatility in these markers. Especially, the three newly developed markers (co-dominant PAMSA 67N04_P and SCAR markers PPR621.5R and PPR621.5M) represent valuable tools for marker-assisted selection in sunflower hybrid breeding.

## Figures and Tables

**Table 1:** BAC clones identified by hybridizations against BAC libraries from RHA 325 and HA 383.

Probe	Library	BAC Clone	Probe	Library	BAC Clone
OP-K13_454A	RHA 325	067N04	67N04-BAC end	RHA 325	139A17
		059J13			179K02
		067I05		HA 383	060K10
	HA 383	216F17			089P04
		225D09			153F08
		401E15			177K11
E41M48_113A	RHA 325	094F15			177M08
	HA 383	100L22			178G06
		147A03			180A24
		233O05			192D15
		482D10			312E12
E62M52-249A	HA 383	006N12			467E16
		447N06			486L01
		480G04	E44M70_275A	HA383	450B06

**Table 2:** Potential candidate genes for the restorer gene *Rf1* on chromosome 13.

Gene ID	Gene Annotation	Orientation	chrom Start	chrom End	cds Start	cds Stop	Length Total
HanXRQChr13g0392791	Putative pentatricopeptide repeat	+	41353989	41357247	41355989	41356747	3258
HanXRQChr13g0393411	Probable aldehyde dehydrogenase 22A1	+	45380421	45389613	45382421	45389113	9192
HanXRQChr13g0394161	Probable pentatricopeptide repeat (PPR) superfamily protein	-	51625496	51621365	51623496	51621865	4131
HanXRQChr13g0394751	Probable poly(A) polymerase 3 (PAPS3)	-	56435098	56428877	56433098	56429377	6221
HanXRQChr13g0418841	Putative pentatricopeptide repeat	+	170848155	170852502	170850155	170852002	4347
HanXRQChr13g0418851	Putative tetratricopeptide-like helical domain	+	170875322	170879807	170877322	170879307	4485
HanXRQChr13g0418861	Putative pentatricopeptide repeat	+	170906019	170909610	170908019	170909110	3591
HanXRQChr13g0419621	PP198, Pentatricopeptide repeat	-	173477525	173472987	173475525	173473487	4538
HanXRQChr13g0419631	Putative pentatricopeptide repeat	+	173482455	173500901	173484455	173500401	18446

Orange: region between 28,051,124 bp (067N04_SP6)–58,081,625 (89P04_T7). White: region between 169,655,088 bp (ORS1030)–173,581,392 bp (OP-H13).

**Table 3:** Overview of the SNPs and INDELs detected in the candidate genes for *Rf1* detected in an association panel of 27 maintainer lines and 32 restorer lines (Minor allele frequency > 5%).

Gene ID	Short Name	Total Variants	Whole Genome Area (Including 2000 bp Upstream and 500 bp Downstream of the Gene)		Exon	
			No. SNPs	No. INDELs	No. SNPs	No. INDELs
HanXRQChr13g0392791	PPR791	7	-	7	-	-
HanXRQChr13g0393411	ALD22A1	9	-	9	-	6
HanXRQChr13g0394161	PPR161	-	-	-	-	-
HanXRQChr13g0394751	PAPS3	7	4	3	2	2
HanXRQChr13g0418841	PPR841	46	36	10	22	3
HanXRQChr13g0418851	TPR851	56	45	11	24	4
HanXRQChr13g0418861	PPR861	30	24	6	1	-
HanXRQChr13g0419621	PPR621	30	26	4	22	1
HanXRQChr13g0419631	PPR631	92	75	17	7	-

Orange: region between 28,051,124 bp (067N04_SP6)–58,081,625 (89P04_T7). White: region between 169,655,088 bp (ORS1030)–173,581,392 bp (OP-H13).

**Table 4:** Significant association of SNPs with restoration of fertility at *p*-value < 10^−3^.

SNP	Chromosome *	Position of SNP *	SNP_F	*p*-Value
PPR861.11	13	170,907,603	26.37	3.68 × 10^−6^
PPR861.3	13	170,906,233	15.83	3.88 × 10^−6^
PPR861.19	13	170,908,139	25.94	4.61 × 10^−6^
PPR841.29	13	170,851,469	22.23	1.65 × 10^−5^
PPR841.38	13	170,851,758	22.23	1.65 × 10^−5^
PPR841.26	13	170,851,288	21.5	2.22 × 10^−5^
PPR861.9	13	170,907,279	12.42	3.53 × 10^−5^
PPR621.5	13	173,473,513	18.25	7.73 × 10^−5^
PPR621.11	13	173,473,976	18.25	7.73 × 10^−5^
PPR841.39	13	170,851,781	18.01	8.34 × 10^−5^
PPR861.20	13	170,908,143	10.02	2.00 × 10^−4^
PPR841.40	13	170,851,807	10.07	2.76 × 10^−4^
PPR621.27	13	173,475,377	14.88	2.98 × 10^−4^
PPR631.79	13	173,498,360	14.88	2.98 × 10^−4^
PPR861.26	13	170,908,690	9.28	3.36 × 10^−4^
TPR851.2	13	170,875,633	8.93	4.65 × 10^−4^
PPR621.10	13	173,473,934	13.25	6.02 × 10^−4^
PPR621.4	13	173,473,493	13.25	6.02 × 10^−4^
PPR621.30	13	173,475,599	8.64	8.08 × 10^−4^
PPR861.27	13	170,909,093	8.36	8.39 × 10^−4^
PPR621.9	13	173,473,774	12.38	9.01 × 10^−4^

* Position of SNP in accordance with the sunflower genome sequence HanXRQ.

**Table 5:** Haplotypes based on the 10 significantly with fertility restoration associated SNPs.

Genotypes	PPR 841.26	PPR 841.29	PPR 841.38	PPR 841.39	PPR 861.3	PPR 861.9	PPR 861.11	PPR 861.19	PPR 621.5	PPR 621.11	Percentage
HanXRQ	TT	GG	GG	CC	GG	AA	CC	GG	GG	GG	
Maintainer	TT	GG	GG	CC	GG	AA	CC	GG	GG	GG	85.2%
Restorer	CC	GA	AA	CT	AA	TT	CT	CC	CC	--	56.3%
Restorer	CC	GA	AA	CY	RA	WT	CT	CC	CC	--	81.3%

**Table 6:** Specificity of molecular markers used for detection of fertility restoration in the sunflower association panel.

Marker	Type of Marker	No. of Lines with Restorer Profile	No. of Lines with Maintainer Profile	No. of Heterozygous Lines	No. of Lines with Data
HRG01 (K13)	Dominant	71	486	-	557
HRG02 (Y10)	Dominant	127	430	-	557
orfH522-CMS	Dominant	135	422	-	557
H13 *Hinf*I	Co-dominant	71	332	6	409
67N04_P	Co-dominant	130	426	19	556
PPR621.5R	Dominant	114	409	24	547
PPR621.5M	Dominant	114	409	24	547

## Supplementary Materials

Supplementary materials can be found at https://www.mdpi.com/1422-0067/20/6/1260/s1. Supplementary Table S1: Plant material of the association panel (59 accessions) for the NGS approach. Supplementary Table S2: Haplotype variation revealed by SNP analyses in the two potential target regions for *Rf1*, the 30-Mb-region (28,051,124-58,081,625), and the 3.9-Mb-region (169,655-173,582,392) on chromosome 13 in a set of 59 sunflower lines. Supplementary Table S3: Large association panel (557 accessions; M = maintainer, R = restorer) with screened marker information for *Rf1*. Supplementary Table S4: Overgo primers used in colony hybridization against two BAC libraries. Supplementary Table S5: SSR primer combinations used for analyses of population structure. Supplementary Table S6: Primer combinations of the newly developed SCAR and PAMSA markers, as well as other primers used in the publication. Supplementary Table S7: Read counts for the SNPs of the 59 genotypes by next generation sequencing of nine candidate genes for Rf1.
