# Supplementary material for: Development and Validation of Markers for the Fertility Restorer Gene Rf1 in Sunflower
Source: Int J Mol Sci. 2019 Mar 13;20(6):1260. doi: 10.3390/ijms20061260 (PMC6471545; doi:10.3390/ijms20061260)
Supplement: Supplementary file 1 [file ijms-20-01260-s001.zip › Supplementary Table 1, 3, 4, 5, 6.pdf]

Supplementary Table 1: Plant material of the association panel (59 accessions) for the NGS approach

Accession ID	Common name	Common name	R/M
CN 36719	54-3	54-3	M
CN42283	Armavirsky 3497	Armavirsky 3497	M
PI 650649	Arrowhead	Arrowhead	M
CN 36594	CM104	CM104	M
CN36654	CM259	CM259	M
CN36665	CM275	CM275	M
CN37369	CM526	CM526	M
CN36562	CM63	CM63	M
CN33280	Krasnodarets	Krasnodarets	M
PI 221693	No. 2	No. 2	M
PI 650497	ROMSUN V3355	ROMSUN V3355	M
Ames 31746	UGA-SAM1-053	HA 412 HO	M
Ames 31775	UGA-SAM1-082	ND-NONOIL B2	M
Ames 31801	UGA-SAM1-109	ND-EBLYS	M
Ames 31802	UGA-SAM1-110	HA 318	M
Ames 31832	UGA-SAM1-140	HA 394	M
Ames 31845	UGA-SAM1-153	HA 433	M
Ames 31847	UGA-SAM1-155	HA 446	M
Ames 31848	UGA-SAM1-156	HA 65	M
Ames 31863	UGA-SAM1-171	HA 61	M
Ames 31875	UGA-SAM1-183	HA 207	M
Ames 31877	UGA-SAM1-185	HA 821	M
Ames 31911	UGA-SAM1-219	HA 1	M
Ames 31916	UGA-SAM1-224	HA 248	M
Ames 31956	UGA-SAM1-264	INRA line SF_233	M
Ames 31965	UGA-SAM1-273	INRA line SF_076	M
PI 431542	D-75-10	D-75-10	M
PI 432519	GN 0778	GN 0778	R
CN 42267	RHA 282	RHA 282	R
Ames 31703	UGA-SAM1-010	RHA 377	R
Ames 31705	UGA-SAM1-012	RHA 381	R
Ames 31717	UGA-SAM1-024	RHA 273	R
Ames 31734	UGA-SAM1-041	RGIG1	R
Ames 31748	UGA-SAM1-055	RHA 464	R
Ames 31750	UGA-SAM1-057	RHA 855	R
Ames 31792	UGA-SAM1-100	RHA 330	R
Ames 31793	UGA-SAM1-101	RHA 331	R
Ames 31810	UGA-SAM1-118	RHA 361	R
Ames 31811	UGA-SAM1-119	RHA 362	R
Ames 31813	UGA-SAM1-121	RHA 367	R
Ames 31828	UGA-SAM1-136	RHA 373	R

Ames 31841	UGA-SAM1-149	HA-R7	R
Ames 31853	UGA-SAM1-161	RHA 395	R
Ames 31857	UGA-SAM1-165	RHA 274	R
Ames 31861	UGA-SAM1-169	RHA 299	R
Ames 31873	UGA-SAM1-181	RHA 311	R
Ames 31878	UGA-SAM1-186	RHA 417	R
Ames 31883	UGA-SAM1-191	RHA 408	R
Ames 31887	UGA-SAM1-195	RHA 418	R
Ames 31890	UGA-SAM1-198	R-190	R
Ames 31891	UGA-SAM1-199	R-206	R
Ames 31896	UGA-SAM1-204	RHA 427	R
Ames 31899	UGA-SAM1-207	RHA 419	R
Ames 31910	UGA-SAM1-218	1972 R-Line Composite	R
Ames 31934	UGA-SAM1-242	RHA 355	R
Ames 31935	UGA-SAM1-243	RHA 366	R
Ames 31968	UGA-SAM1-276	INRA line SF_306	R
Ames 31970	UGA-SAM1-278	INRA line SF_295	R
Ames 31980	UGA-SAM1-288	INRA line SF_323	R

Supplementary Table 3: Large association panel (557 accessions; M=maintainer, R=restorer) with screened marker information for *Rf1*

Accession No.	Common Name 1	Common Name 2	M/R	orfH522-CMS	Y10 Restorer	K13 Restorer	H13 PCR product	H13 HinfI	67N4_P	621.5 F1	621.5 F2
PI 162454	SUNRISE			0	0	0	0		155	1	0
PI 170412	No. 2770			0	0	0	1	42,101,165	155	1	0
PI 170419	No. 3332			0	0	0	1	42,101,165	155	1	0
PI 171655	AYCICEGI			0	0	0	1	42,101,165	155	1	0
PI 175723	GONONDU			0	0	0	1	42,101,165	155	1	0
PI 184048	NOVOSADSKI BR. 4			0	0	0	1	42,101,165	155	1	0
PI 195573	No. 9588			0	0	0	1	42,101,165	155	1	0
PI 213175				0	0	0	1	42,101,165	155	1	0
PI 221441	AFTAB-PARAST			0	0	0	1	42,101,165	155	1	0
PI 221693	No. 2		M	0	0	0	1	42,101,165	155	1	0
PI 232904	LOVASZPATONAI			0	0	0	1	42,101,165	155	1	0
PI 243074	W.Y. I/7			0	0	0	1	42,101,165	155	1	0
PI 251901	JDANOVSKY6432 ND 31%OIL			0	0	0	1	42,101,165	155	1	0
PI 251990	No. K1879			0	0	0	1	42,101,165	155	1	0
PI 256334				0	0	0	0		155	1	0
PI 263178				0	0	0	1	42,101,165	155	1	0
PI 265099	VNIIMK 1646 4 MOTH RES			0	0	0	1	42,101,165	155	1	0
PI 265499	CINZA 42			0	0	0	0		155	1	0
PI 287230	ENISEJ			0	0	0	1	42,101,165	155	1	0
PI 289626	No. 5			0	0	0	1	42,101,165	155	1	0
PI 291404	SZARATOVSZKIJ RANNI			0	0	0	1	42,101,165	155	1	0
PI 296289	JUPITER			0	0	0	1	42,101,165	155	1	0
PI 307831	AGUAPEI			0	0	0	0		155	1	0
PI 307934	ARMAVIRSKY			0	0	0	1	42,101,165	155	1	0
PI 307942	VNIIMK 8883			0	0	0	1	42,101,165	155	1	0
PI 323281	BLACK SAYAR			0	0	0	1	42,101,165	155	1	0
PI 331176	MANFREDI INTA (3-WAY X)11			0	0	0	0		155	0	0
PI 340784	USSR MAYAK '66			0	0	0	1	42,101,165	155	1	0

PI 340790	USSR VNIIMK 8931 '66			0	0	0	1	42,101,165	155	1	0
PI 343798	TCHERNIANKA SELECT W-17			0	0	0	1	42,101,165	155	1	0
PI 343809	W-31			0	0	0	1	42,101,165	155	1	0
PI 369358	HAVASUPAI			0	0	0	1	42,101,165	155	1	0
PI 369359	HOPI			0	0	0	0		155	1	0
PI 369360	SENECA			0	0	0	1	42,101,165	155	1	0
PI 371936	VOSHOD			0	0	0	1	42,101,165	155	1	0
PI 372258	SMENA			0	0	0	1	42,101,165	155	1	0
PI 372259	VNIIMK 6540			0	0	0	1	42,101,165	155	1	0
PI 377528	DARK STRIPE			0	0	0	1	42,101,165	155	1	0
PI 377530	KENYA WHITE			0	0	0	1	42,101,165	155	1	0
PI 378894	GUAYACAN INTA			0	0	0	1	42,101,165	155	1	0
PI 600717	Mandan #1			0	0	0	1	42,101,165	155	1	0
PI 600705	CO-PB 68			0	1	0	1	42,101,165,207	170.155	1	1
PI 596750	Rf TUB-346			1	0	0	1	42,101,165	155	1	0
PI 535894	WIELKOPOLSKI			0	0	0	1	42,101,165	155	1	0
PI 535890	KRZYNOWLOSKI MIEJSCOWY			0	0	0	1	42,101,165	155	1	0
PI 531351	GEORGIA			0	0	0	1	42,101,165	155	1	0
PI 531350	FRANCIA "E"			0	0	0	1	42,101,165	155	1	0
PI 531345	CSEHSZLOVAKIAI "B"			0	0	0	1	42,101,165	155	1	0
PI 531339	BEKECSI "B"			0	0	0	1	42,101,165	155	1	0
PI 507901	3100404			0	0	0	1	42,101,165	155	1	0
PI 507899	3100402			0	0	0	1	42,101,165	155	1	0
PI 505839	Odesskij 113			1	1	0	0		170	0	1
PI 500688				0	0	0	1	42,101,165	155	1	0
PI 497939	VK-12			0	0	0	1	42,101,165	155	1	0
PI 497937	Start			0	1	0	1	42,101,165	170	1	1
PI 497250	VIR 160			0	0	0	1	101,207	155	1	0
PI 497247	VIR 019			0	0	0	1	42,101,165	155	1	0
PI 496265	Liao 2			0	0	0	1	42,101,165	155	1	0
PI 496263	Damaya			0	0	0	0		155	1	0

PI 490324	Ames 2350			0	0	0	1	42,101,165	155	1	0
PI 490281	Ames 101			0	0	0	1	42,101,165	155	1	0
PI 487194	Egnazia			0	0	0	1	42,101,165	155	1	0
PI 483077	Pervenets			0	0	0	1	42,101,165	155	1	0
PI 480472	CCA82-2			1	1	1	1	42,101,165	170.155	1	1
PI 433377	Giza			0	0	0	1	42,101,165	155	1	0
PI 432519	GN 0778			1	1	0	1	42,101,165	170	1	1
PI 432512	GN 0778			1	0	0	1	42,101,165	155	1	0
PI 431558	NS-B-16-63/1			0	0	0	1	42,101,165,207	155	1	0
PI 431542	D-75-10		M	0	0	0	1	101,207	155	1	0
PI 431538	D-75-4			0	0	0	0		155	1	0
PI 431529	43-48 VK-32			0	0	0	1	42,101,165	155	1	0
PI 431516	Romsun N-2-2004			0	0	0	0		155	1	0
PI 430541	PROGRESS			0	0	0	1	42,101,165	155	1	0
PI 430539	CAKINSKIJ 321			0	0	0	1	42,101,165	155	1	0
PI 424926	KORTUS			0	0	0	1	42,101,165	155	1	0
PI 408726	RELAX HYBRID GERMPLASM			1	0	0	1	42,101,165	155	1	0
PI 386230	VIR 847			0	0	0	0		155	1	0
PI 386096	HATZOR AYALA			0	0	0	1	42,101,165	155	1	0
PI 380576	ARGE PEHUEN			0	0	0	1	42,101,165	155	1	0
PI 378895	IMPIRA INTA			0	0	0	1	42.101.165	155	1	0
PI 600721	Hidatsa #2			0	0	0	1	42,101,165	155	1	0
PI 617026	BR1			0	0	0	1	42,101,165	155	1	0
PI 617027	BR2			0	0	0	1	42,101,165	155	1	0
PI 617029	BR4			0	0	0	1	42,101,165	155	1	0
PI 650337	Hemas			0	0	0	1	42,101,165	155	1	0
PI 650343	Record			0	0	0	1	42,101,165	155	1	0
PI 650344	Sepasol			0	0	0	1	42,101,165	155	1	0
PI 650350	L-2625-1(Ukraine)-1			0	0	0	1	42,101,165	155	1	0
PI 650362	6 SC UG L6			1	0	0	0		155	1	0
PI 650370	Oleisty Borovskij			0	0	0	1	42,101,165	155	1	0

PI 650391	B4268			0	0	0	1	42,101,165	155	0	0
PI 650406	Fuksinka 10			0	0	0	0		155	1	0
PI 650407	Gigant 549			0	0	0	1	42,101,165	155	1	0
PI 650413	Kvuglik A-41			0	0	0	1	42,101,165	155	1	0
PI 650415	B-7422			0	1	0	0		170.155	1	1
PI 650420	Yawne			0	0	0	1	42,101,165	155	1	0
PI 650438	Advance			0	0	0	1	42,101,165	155	1	0
PI 650467	VK-47			0	0	0	1	42,101,165	155	1	0
PI 650472	HS 61			0	0	0	1	42,101,165	155	1	0
PI 650497	ROMSUN V3355		M	0	0	0	1	42,101,165	155	1	0
PI 650530	371-3 S			0	0	0	0		155		
PI 650534	VIR 107			0	0	0	1	101,207	155	1	0
PI 650558	Karlik			0	0	0	1	42,101,165	155	1	0
PI 650649	Arrowhead		M	0	0	0	1	42,101,165	155	1	0
PI 650650	Mennonite			0	0	0	1	42,101,165	155	1	0
PI 650655	Chang Ling			0	0	0	1	42,101,165	155	1	0
PI 650657	JB 4			0	0	0	1	42,101,165	155	1	0
PI 650727	Sannace			0	0	0	0		155	1	0
PI 650731	Stepnyak			0	0	0	1	42,101,165	155	1	0
PI 650735	Zaria			0	0	0	1	42,101,165	155	1	0
PI 650741	Skorospelyi			0	0	0	1	42,101,165	155	1	0
PI 650781	Kosim			0	0	0	1	42,101,165	155	1	0
PI 650788	Ames 21671			0	0	0	1	42,101,165	155	1	0
PI 653587	SAE-005			0	0	0	0		155	1	0
PI 664140	Sundak			0	1	0	1	42,101,165	170	1	1
PI 664194	ND-NONOIL B3			0	0	0	1	42,101,165	155	1	0
PI 664198	ND-NONOIL M2			0	0	0	1	42,101,165	155	1	0
Ames 31694	UGA-SAM1-001	HA 851	M	0	0	0	1	42,101,165	155	1	0
Ames 31695	UGA-SAM1-002	HA 853	M	0	0	0	1	42,101,165	155	1	0
Ames 31696	UGA-SAM1-003	HA 323	M	0	0	0	0		155	1	0
Ames 31736	UGA-SAM1-043	HA-R3	M	0	0	0	1	42,101,165	155	1	0

Ames 31735	UGA-SAM1-042	RHA 801	R	1	1	1	1	101,207	170	0	1
Ames 31734	UGA-SAM1-041	RGIG1	R	0	1	1	0		170	0	1
Ames 31733	UGA-SAM1-040	HA 370	M	0	0	0	1	42,101,165	155	1	0
Ames 31732	UGA-SAM1-039	HA 350	M	0	0	0	1	42,101,165	155	1	0
Ames 31731	UGA-SAM1-038	HA 113	M	0	0	0	1	42,101,165	155	1	0
Ames 31730	UGA-SAM1-037	HA 133	M	0	0	0	1	42,101,165	155	1	0
Ames 31729	UGA-SAM1-036	HA 116	M	0	0	0	1	42,101,165	155	1	0
Ames 31728	UGA-SAM1-035	HA 112	M	0	0	0	0		155	1	0
Ames 31727	UGA-SAM1-034	RHA 437	R	1	0	0	1	42,101,165	155	1	0
Ames 31726	UGA-SAM1-033	RHA 436	R	1	0	0	0		155	1	0
Ames 31725	UGA-SAM1-032	R-201	R	0	0	0	1	101,207	155	1	0
Ames 31724	UGA-SAM1-031	R-188	R	0	1	0	0		170	0	1
Ames 31723	UGA-SAM1-030	R-185	R	0	1	1	1	101,207	170	0	1
Ames 31722	UGA-SAM1-029	HA 313	M	0	0	0	1	42,101,165	155	1	0
Ames 31721	UGA-SAM1-028	HA 306	M	0	0	0	0		155	1	0
Ames 31720	UGA-SAM1-027	HA 314	M	0	0	0	1	42,101,165	155	1	0
Ames 31719	UGA-SAM1-026	HA 124	M	0	0	0	1	42,101,165	155	1	0
Ames 31718	UGA-SAM1-025	RHA 298	R	1	1	1	1	101,207	170	0	1
Ames 31717	UGA-SAM1-024	RHA 273	R	1	1	1	1	101,207	170	0	1
Ames 31716	UGA-SAM1-023	RHA 397	R	1	0	0	0		155	1	0
Ames 31715	UGA-SAM1-022	RHA 396	R	1	1	1	1	101,207	170	0	1
Ames 31714	UGA-SAM1-021	HA 405	M	0	0	0	1	42,101,165	155	1	0
Ames 31713	UGA-SAM1-020	HA 404	M	0	0	0	1	42,101,165	155	1	0
Ames 31712	UGA-SAM1-019	HA 403	M	0	0	0	1	42,101,165	155	1	0
Ames 31711	UGA-SAM1-018	HA 384	M	0	0	0	1	42,101,165	155	1	0
Ames 31710	UGA-SAM1-017	HA 383	M	0	0	0	1	42,101,165	155	1	0
Ames 31709	UGA-SAM1-016	RHA 389	R	1	1	0	0		170	0	1
Ames 31708	UGA-SAM1-015	RHA 388	R	1	1	1	0		170	0	1
Ames 31707	UGA-SAM1-014	RHA 387	R	1	1	1	0		170	0	1
Ames 31706	UGA-SAM1-013	RHA 386	R	1	1	1	0		170	0	1
Ames 31705	UGA-SAM1-012	RHA 381	R	1	1	1	0		170	0	1

Ames 31704	UGA-SAM1-011	HA 380	M	0	0	0	1	42,101,165	155	1	0
Ames 31703	UGA-SAM1-010	RHA 377	R	1	1	0	1	101,207	170	0	1
Ames 31702	UGA-SAM1-009	RHA 376	R	1	1	1	1	101,207	170	0	1
Ames 31701	UGA-SAM1-008	RHA 280	R	0	0	0	1	42,101,165	155	1	0
Ames 31700	UGA-SAM1-007	HA 302	M	0	0	0	1	42,101,165	155	1	0
Ames 31699	UGA-SAM1-006	HA 286	M	0	0	0	0		155	1	0
Ames 31698	UGA-SAM1-005	HA 352	M	0	0	0	1	42,101,165	155	1	0
Ames 31697	UGA-SAM1-004	HA 351	M	0	1	1	1	42,101,165	170	1	1
Ames 31737	UGA-SAM1-044	VK-47		0	0	0	0		155	1	0
Ames 31738	UGA-SAM1-045	ND-BLPL2		0	0	0	1	42,101,165	155	1	0
Ames 31739	UGA-SAM1-046	Mandan #1		0	0	0	1	42,101,165	155	1	0
Ames 31740	UGA-SAM1-047	HA 452	M	0	1	0	0		170	1	1
Ames 31741	UGA-SAM1-048	Klein Casares		0	0	0	1	42,101,165	155	1	0
Ames 31742	UGA-SAM1-049	RHA 455	R	1	1	1	0		170	0	1
Ames 31743	UGA-SAM1-050	HA 456	M	0	0	0	1	42,101,165	155	1	0
Ames 31744	UGA-SAM1-051	USSR VNIIMK 8931 '66		0	0	0	1	42,101,165	155	1	0
Ames 31745	UGA-SAM1-052	HA 457	M	0	0	0	1	42,101,165	155	1	0
Ames 31746	UGA-SAM1-053	HA 412 HO	M	0	0	0	1	42,101,165	155	1	0
Ames 31747	UGA-SAM1-054	RHA 463	R	1	1	1	1	101,207	170	0	1
Ames 31748	UGA-SAM1-055	RHA 464	R	0	1	1	1	101,207	170	0	1
Ames 31749	UGA-SAM1-056	RHA 324	R	1	1	1	1	101,207	170	0	1
Ames 31750	UGA-SAM1-057	RHA 855	R	1	1	0	1	101,207	170	0	1
Ames 31751	UGA-SAM1-058	ND-ERLYS		0	1	0	0		170	0	1
Ames 31752	UGA-SAM1-059	HA 349	M	0	0	0	0		155	1	0
Ames 31753	UGA-SAM1-060	RHA 359	R	1	1	0	0		170	0	1
Ames 31754	UGA-SAM1-061	RHA 364	R	1	0	0	1	42,101,165	155	0	1
Ames 31755	UGA-SAM1-062	HA 371	M	0	0	0	1	42,101,165	155	1	0
Ames 31756	UGA-SAM1-063	HA GERMPLASM POOL III-G		0	0	0	1	42,101,165	155	1	0
Ames 31757	UGA-SAM1-064	HA GERMPLASM POOL III-Q		1	1	0	1	42,101,165	170.155	1	1
Ames 31758	UGA-SAM1-065	RHA 296	R	1	1	1	1	101,207	170	0	1
Ames 31759	UGA-SAM1-066	DM-3		0	0	0	1	42,101,165	155	1	0

Ames 31760	UGA-SAM1-067	HA 378	M	0	0	0	1	42,101,165	155	1	0
Ames 31761	UGA-SAM1-068	HA 154	M	0	0	0	0		170	0	1
Ames 31762	UGA-SAM1-069	HA 442	M	0	0	0	1	42,101,165	155	1	0
Ames 31763	UGA-SAM1-070	HA 285	M	0	0	0	1	42,101,165	155	1	0
Ames 31764	UGA-SAM1-071	HA 289	M	0	0	0	1	42,101,165	155	1	0
Ames 31765	UGA-SAM1-072	RHA 309	R	1	1	0	0		170	0	1
Ames 31766	UGA-SAM1-073	PHA009		1	1	0	1	101,207	170	0	1
Ames 31767	UGA-SAM1-074	HA 228	M	0	0	0	1	42,101,165	155	1	0
Ames 31768	UGA-SAM1-075	HA GERMPLASM POOL I		0	0	0	1	42,101,165	155	1	0
Ames 31769	UGA-SAM1-076	HA 312	M	0	0	0	1	42,101,165	155	1	0
Ames 31770	UGA-SAM1-077	HA 850	M	0	1	0	1	42,101,165	170	1	1
Ames 31771	UGA-SAM1-078	HA 322	M	0	0	0	1	42,101,165	155	1	0
Ames 31772	UGA-SAM1-079	HA GERMPLASM POOL III-B		0	0	0	1	42,101,165	155	1	0
Ames 31773	UGA-SAM1-080	HA 287	M	1	0	0	1	42,101,165	155	1	0
Ames 31774	UGA-SAM1-081	RHA 375	R	1	1	1	1	101,207	170	0	1
Ames 31775	UGA-SAM1-082	ND-NONOIL B2		0	0	0	1	42,101,165	155	1	0
Ames 31776	UGA-SAM1-083	Hopi dye		0	0	0	0		155	1	1
Ames 31777	UGA-SAM1-084	HA GERMPLASM POOL II		0	0	0	1	42,101,165	155	1	0
Ames 31778	UGA-SAM1-086	HA GERMPLASM POOL V-1I		0	0	0	1	42,101,165	155	1	0
Ames 31779	UGA-SAM1-087	HA GERMPLASM POOL V-1K		1	0	0	1	42,101,165	170	1	1
Ames 31780	UGA-SAM1-088	HA GPP V-2		0	0	0	1	42,101,165	155	1	0
Ames 31781	UGA-SAM1-089	ND-NONOIL 2		0	0	0	1	42,101,165	155	1	0
Ames 31782	UGA-SAM1-090	HA 458	M	0	0	0	1	42,101,165	155	1	0
Ames 31783	UGA-SAM1-091	ND-NONOIL 3		0	0	0	1	42,101,165	155	1	0
Ames 31784	UGA-SAM1-092	ND-NONOIL B3		0	0	0	1	42,101,165	155	1	0
Ames 31785	UGA-SAM1-093	VIR 847		0	0	0	0		155	1	0
Ames 31786	UGA-SAM1-094	Mammoth		0	0	0	1	42,101,165	155	1	0
Ames 31787	UGA-SAM1-095	ND-NONOIL B5		0	0	0	1	42,101,165	155	1	0
Ames 31788	UGA-SAM1-096	RHA 462	R	1	1	0	1	101,207	170	0	1
Ames 31789	UGA-SAM1-097	ND-NONOIL M3		0	0	0	0		155	1	0
Ames 31790	UGA-SAM1-098	RHA 326	R	0	0	0	1	42,101,165	155	1	1

Ames 31791	UGA-SAM1-099	RHA 329	R	1	0	0	1	101,207	170	0	1
Ames 31792	UGA-SAM1-100	RHA 330	R	1	1	1	1	101,207	170	0	1
Ames 31793	UGA-SAM1-101	RHA 331	R	1	1	1	1	101,207	170	0	1
Ames 31794	UGA-SAM1-102	RHA 332	R	1	1	1	1	101,207	170	0	1
Ames 31795	UGA-SAM1-103	RHA 333	R	1	1	1	1	101,207	170	0	1
Ames 31796	UGA-SAM1-104	HA 852	M	0	0	0	1	42,101,165	155	1	0
Ames 31797	UGA-SAM1-105	ND-BLOS		0	0	0	0		155	1	0
Ames 31798	UGA-SAM1-106	RHA 854	R	1	1	1	1	101,207	170	0	1
Ames 31799	UGA-SAM1-107	RHA 857	R	1	1	1	1	101,207	170.155	0	1
Ames 31800	UGA-SAM1-108	RHA 858	R	1	1	1	1	101,207	170	0	1
Ames 31801	UGA-SAM1-109	ND-EBLYS		0	0	0	1	42,101,165	155	1	0
Ames 31802	UGA-SAM1-110	HA 318	M	0	0	0	1	42,101,165	155	1	0
Ames 31803	UGA-SAM1-111	HA 319	M	0	0	0	1	42,101,165	155	1	0
Ames 31804	UGA-SAM1-112	HA 320	M	0	0	0	1	42,101,165	155	1	0
Ames 31805	UGA-SAM1-113	HA 321	M	0	0	0	0		155	1	0
Ames 31806	UGA-SAM1-114	RHA 325	R	1	1	1	1	101,207	170	0	1
Ames 31807	UGA-SAM1-115	RHA 347	R	0	0	0	0			0	1
Ames 31808	UGA-SAM1-116	HA 353	M	0	0	0	1	42,101,165	155	1	0
Ames 31809	UGA-SAM1-117	RHA 354	R	1	1	1	1	101.207	170	0	1
Ames 31810	UGA-SAM1-118	RHA 361	R	1	1	1	0		170.155	0	1
Ames 31811	UGA-SAM1-119	RHA 362	R	1	1	1	0		170.155	0	1
Ames 31812	UGA-SAM1-120	RHA 363	R	1	1	1	0		170	0	1
Ames 31813	UGA-SAM1-121	RHA 367	R	1	1	1	1	101.207	170	0	1
Ames 31814	UGA-SAM1-122	RPET2	R	0	1	1	0		170	0	1
Ames 31815	UGA-SAM1-123	HA GERMPLASM POOL III-C		0	0	0	1	42,101,165	155	1	0
Ames 31816	UGA-SAM1-124	HA GERMPLASM POOL III-D		0	0	0	1	42,101,165	155	1	0
Ames 31817	UGA-SAM1-125	HA GERMPLASM POOL III-I		1	1	0	1	42,101,165	170	1	1
Ames 31818	UGA-SAM1-126	HA GERMPLASM POOL III-L		0	0	0	1	42,101,165	155	1	0
Ames 31819	UGA-SAM1-127	HA GERMPLASM POOL III-O		0	0	0	1	42,101,165	155	1	0
Ames 31820	UGA-SAM1-128	HA GERMPLASM POOL III-U		0	0	0	1	42,101,165	155	1	0
Ames 31821	UGA-SAM1-129	HA 288	M	0	0	0	1	42,101,165	155	1	0

Ames 31822	UGA-SAM1-130	HA 290	M	0	0	0	1	42,101,165	155	1	0
Ames 31823	UGA-SAM1-131	HA 291	M	1	0	0	1	42,101,165	155	1	0
Ames 31824	UGA-SAM1-132	HA 292	M	0	0	0	1	42,101,165	155	1	0
Ames 31825	UGA-SAM1-133	HA 300	M	0	0	0	1	42,101,165	155	1	0
Ames 31826	UGA-SAM1-134	HA 305	M	0	0	0	1	42,101,165	155	1	0
Ames 31828	UGA-SAM1-136	RHA 373	R	1	1	1	1	101.207	170	0	1
Ames 31829	UGA-SAM1-137	RHA 374	R	1	1	0	0		170	0	1
Ames 31830	UGA-SAM1-138	HA 385	M	0	0	0	1	42,101,165	155	1	0
Ames 31831	UGA-SAM1-139	HA 393	M	0	0	0	0		155	1	0
Ames 31832	UGA-SAM1-140	HA 394	M	0	0	0	1	42,101,165	155	1	0
Ames 31833	UGA-SAM1-141	HA 406	M	0	0	0	1	42,101,165	155	1	0
Ames 31834	UGA-SAM1-142	RHA 398	R	1	0	0	0		155	1	0
Ames 31835	UGA-SAM1-143	RHA 270	R	0	1	1	0		170	0	1
Ames 31836	UGA-SAM1-144	HA 60	M	0	0	0	1	101.207	155	1	0
Ames 31837	UGA-SAM1-145	HA 277	M	0	0	0	1	42,101,165	155	1	0
Ames 31838	UGA-SAM1-146	RHA 293	R	1	1	1	0		170	0	1
Ames 31839	UGA-SAM1-147	HA 308	M	0	1	1	1	101,207	170	0	1
Ames 31840	UGA-SAM1-148	RHA 391	R	1	1	1	0		170	0	1
Ames 31841	UGA-SAM1-149	HA-R7	R	1	1	1	1	101,207	170.155	0	1
Ames 31842	UGA-SAM1-150	IMISUN-1	M	0	0	0	1	42,101,165	155	1	0
Ames 31843	UGA-SAM1-151	HA 425	M	0	0	0	1	42,101,165,207	155	1	0
Ames 31844	UGA-SAM1-152	HA 429	M	0	0	0	1	42,101,165	155	1	0
Ames 31845	UGA-SAM1-153	HA 433	M	0	0	0	1	42,101,165	155	1	0
Ames 31846	UGA-SAM1-154	RHA 438	R	1	0	0	1	42,101,165	155	1	0
Ames 31847	UGA-SAM1-155	HA 446	M	0	0	0	1	42,101,165	155	1	0
Ames 31848	UGA-SAM1-156	HA 65	M	0	0	0	0		155	1	0
Ames 31849	UGA-SAM1-157	HA 236	M	0	0	0	0		155	1	0
Ames 31850	UGA-SAM1-158	HA 249	M	0	0	0	1	42,101,165	155	1	0
Ames 31852	UGA-SAM1-160	HA 407	M	1	0	0	1	42,101,165	155	1	0
Ames 31853	UGA-SAM1-161	RHA 395	R	1	1	0	0		170	0	1
Ames 31854	UGA-SAM1-162	RHA 399	R	1	1	0	0		170	0	1

Ames 31855	UGA-SAM1-163	RHA 400	R	0	1	1	0		170	0	1
Ames 31856	UGA-SAM1-164	RHA 401	R	0	1	0	1	101.207	170.155	0	1
Ames 31857	UGA-SAM1-165	RHA 274	R	1	1	0	0		170	0	1
Ames 31858	UGA-SAM1-166	RHA 279	R	1	1	0	0		170	0	1
Ames 31859	UGA-SAM1-167	RHA 294	R	1	1	1	1	101.207	170	0	1
Ames 31860	UGA-SAM1-168	RHA 297	R	1	1	1	0		170	0	1
Ames 31861	UGA-SAM1-169	RHA 299	R	1	1	1	0		170	0	1
Ames 31862	UGA-SAM1-170	HA 8	M	0	0	0	0		155	1	0
Ames 31863	UGA-SAM1-171	HA 61	M	0	0	0	1	101.207	155	1	0
Ames 31864	UGA-SAM1-172	HA 64	M	0	0	0	0		155	1	0
Ames 31865	UGA-SAM1-173	HA 89	M	0	0	0	1	42,101,165	155	1	0
Ames 31866	UGA-SAM1-174	HA 99	M	0	0	0	1	42,101,165	155	1	0
Ames 31867	UGA-SAM1-175	HA 224	M	0	0	0	0		155	1	0
Ames 31868	UGA-SAM1-176	HA 234	M	0	0	0	1	42,101,165	155	1	0
Ames 31869	UGA-SAM1-177	HA 304	M	0	0	0	1	42,101,165	155	1	0
Ames 31870	UGA-SAM1-178	HA 822	M	0	0	0	1	42,101,165	155	1	0
Ames 31871	UGA-SAM1-179	RHA 271	R	1	1	1	0		170	0	1
Ames 31872	UGA-SAM1-180	RHA 272	R	1	1	1	1	101.207	170	0	1
Ames 31873	UGA-SAM1-181	RHA 311	R	1	1	1	1	101.207	170	0	1
Ames 31874	UGA-SAM1-182	HA 307	M	0	0	0	1	42,101,165	155	1	0
Ames 31875	UGA-SAM1-183	HA 207	M	0	0	0	1	42,101,165	155	1	0
Ames 31876	UGA-SAM1-184	RHA 310	R	1	1	1	1	101.207	170	0	1
Ames 31877	UGA-SAM1-185	HA 821	M	0	0	0	1	42,101,165	155	1	0
Ames 31878	UGA-SAM1-186	RHA 417	R	0	1	1	0		170.155	0	1
Ames 31879	UGA-SAM1-187	BRS-1		0	0	0	0		155	1	0
Ames 31880	UGA-SAM1-188	BRS-3		0	0	0	1	42,101,165	155	1	0
Ames 31881	UGA-SAM1-189	HA 390	M	0	0	0	0		155	1	0
Ames 31882	UGA-SAM1-190	RHA 392	R	1	1	1	0		170.155	0	1
Ames 31883	UGA-SAM1-191	RHA 408	R	1	1	1	0		170	0	1
Ames 31884	UGA-SAM1-192	RHA 409	R	1	1	1	0		170	0	1
Ames 31885	UGA-SAM1-193	HA 410	M	0	0	0	1	42,101,165	155	1	0

Ames 31886	UGA-SAM1-194	HA 413	M	0	0	0	1	42,101,165	155	1	0
Ames 31887	UGA-SAM1-195	RHA 418	R	0	1	1	0		170	0	1
Ames 31888	UGA-SAM1-196	HA-R6	M	0	0	0	0		155	1	0
Ames 31889	UGA-SAM1-197	HA-R8	M	1	1	1	1	101.207	170	0	1
Ames 31890	UGA-SAM1-198	R-190	R	0	1	1	0		170	0	1
Ames 31891	UGA-SAM1-199	R-206	R	0	0	0	1	42,101,165	155	1	0
Ames 31892	UGA-SAM1-200	IMISUN-2	R	1	1	1	1	101.207	170	0	1
Ames 31893	UGA-SAM1-201	IMISUN-3	M	0	0	0	1	42,101,165	155	1	0
Ames 31894	UGA-SAM1-202	IMISUN-4	R	0	1	1	1	101.207	170.155	0	1
Ames 31895	UGA-SAM1-203	RHA 426	R	1	1	1	0		170	0	1
Ames 31896	UGA-SAM1-204	RHA 427	R	1	1	1	0		170	0	1
Ames 31897	UGA-SAM1-205	HA 421	M	0	0	0	1	42,101,165	155	1	0
Ames 31898	UGA-SAM1-206	HA 422	M	0	0	0	1	42,101,165	155	1	0
Ames 31899	UGA-SAM1-207	RHA 419	R	1	1	0	1	101.207	170	0	1
Ames 31900	UGA-SAM1-208	RHA 428	R	1	0	0	1	101.207	155	0	0
Ames 31901	UGA-SAM1-209	HA 430	M	0	1	0	0		170	0	1
Ames 31902	UGA-SAM1-210	HA 431	M	0	0	0	1	42,101,165	155	1	0
Ames 31903	UGA-SAM1-211	HA 432	M	0	1	0	0		170	0	1
Ames 31904	UGA-SAM1-212	HA 434	M	0	0	0	1	42,101,165	155	1	0
Ames 31905	UGA-SAM1-213	HA 435	M	1	1	0	1	101,207	170	0	1
Ames 31906	UGA-SAM1-214	RHA 439	R	1	1	0	1	101,207	170	0	1
Ames 31907	UGA-SAM1-215	RHA 440	R	1	1	0	0		170	0	1
Ames 31908	UGA-SAM1-216	HA 441	M	0	0	0	0		155	1	0
Ames 31909	UGA-SAM1-217	RHA 443	R	1	1	1	1	101.207	170	0	1
Ames 31910	UGA-SAM1-218	1972 R-Line Composite	R	1	1	0	0		170	0	1
Ames 31911	UGA-SAM1-219	HA 1	M	0	0	0	1	42,101,165	155	1	0
Ames 31912	UGA-SAM1-220	HA 15	M	0	1	0	1	42,101,165	170	1	1
Ames 31913	UGA-SAM1-221	HA 66	M	0	0	0	0		155	1	0
Ames 31915	UGA-SAM1-223	HA 243	M	0	0	0	1	42,101,165	155	1	0
Ames 31916	UGA-SAM1-224	HA 248	M	0	0	0	1	42,101,165	155	1	0
Ames 31917	UGA-SAM1-225	HA 253	M	0	0	0	0		155	1	0

Ames 31918	UGA-SAM1-226	HA 259	M	0	0	0	0		155	1	0
Ames 31919	UGA-SAM1-227	HA-R2	M	0	0	0	1	42,101,165	155	1	0
Ames 31920	UGA-SAM1-228	HA-R4	M	0	0	0	0		155	1	0
Ames 31921	UGA-SAM1-229	Sep. HA G POOL VII HMO BULK		0	0	0	1	42,101,165	155	1	0
Ames 31922	UGA-SAM1-230	RHA 334	R	1	1	1	1	101.207	170	0	1
Ames 31923	UGA-SAM1-231	HA 341	M	0	0	0	1	42,101,165	155	0	0
Ames 31924	UGA-SAM1-232	RHA 368	R	1	1	0	1	101.207	170	0	1
Ames 31925	UGA-SAM1-233	HA 379	M	0	0	0	1	42,101,165	155	1	0
Ames 31926	UGA-SAM1-234	HA GERMPLASM POOL V-1J		0	0	0	1	42,101,165	155	1	0
Ames 31927	UGA-SAM1-235	ND-NONOIL 1		0	0	0	1	42,101,165	155	1	0
Ames 31928	UGA-SAM1-236	ND-NONOIL M1		1	1	1	0		170	0	1
Ames 31929	UGA-SAM1-237	RHA 328	R	1	1	0	1	101.207	170	0	1
Ames 31930	UGA-SAM1-238	ND-RLOS		1	1	0	0		170	0	1
Ames 31931	UGA-SAM1-239	RHA 859	R	1	1	0	1	101,207	170	0	1
Ames 31932	UGA-SAM1-240	HA 316	M	0	0	0	1	42,101,165	155	0	0
Ames 31933	UGA-SAM1-241	HA 343	M	0	0	0	1	42,101,165	155	1	0
Ames 31934	UGA-SAM1-242	RHA 355	R	1	1	1	1	101.207	170	0	1
Ames 31935	UGA-SAM1-243	RHA 366	R	1	0	0	0		155	1	0
Ames 31936	UGA-SAM1-244	HA 372	M	0	0	0	1	42,101,165	155	1	0
Ames 31937	UGA-SAM1-245	HA GERMPLASM POOL III-H		1	0	0	1	42,101,165	155	1	0
Ames 31938	UGA-SAM1-246	HA GERMPLASM POOL III-T		1	0	0	1	42,101,165	170.155	1	1
Ames 31939	UGA-SAM1-247	HA 301	M	0	1	0	1	42,101,165	155	1	0
Ames 31940	UGA-SAM1-248	RHA 282	R	1	1	0	0		170	0	1
Ames 31941	UGA-SAM1-249	HA 402	M	0	0	0	1	42,101,165	155	1	0
Ames 31942	UGA-SAM1-250	RHA 278	R	0	0	0	0		155	0	0
Ames 31943	UGA-SAM1-251	RHA 415	R	1	1	0	0		170	0	1
Ames 31944	UGA-SAM1-252	HA 423	M	0	0	0	1	42,101,165	155	1	0
Ames 31945	UGA-SAM1-253	HA 1	M	0	0	0	1	42,101,165	155	1	0
Ames 31946	UGA-SAM1-254	INRA line SF_145	M	0	0	0	0		155	1	0
Ames 31947	UGA-SAM1-255	Manchurian		0	0	0	1	42,101,165	155	1	0
Ames 31948	UGA-SAM1-256	INRA line SF_281	R	1	0	0	1	42,101,165	155	1	0

Ames 31949	UGA-SAM1-257	Guayacan		0	0	0	0		155	1	0
Ames 31950	UGA-SAM1-258	INRA line SF_179	M	0	0	0	1	42,101,165	155	1	0
Ames 31951	UGA-SAM1-259	INRA line SF_092	M	0	0	0	1	42,101,165	155	1	0
Ames 31952	UGA-SAM1-260	Charata		0	0	0	0		155	0	0
Ames 31953	UGA-SAM1-261	INRA line SF_193	M	0	0	0	1	42,101,165	155	1	0
Ames 31954	UGA-SAM1-262	INRA line SF_230	M	1	0	0	0		155	1	1
Ames 31955	UGA-SAM1-263	INRA line SF_232	M	1	0	0	0		155	0	1
Ames 31956	UGA-SAM1-264	INRA line SF_233	M	1	0	0	0		155	1	0
Ames 31957	UGA-SAM1-265	Hemus		0	1	0	1	42,101,165	170	1	1
Ames 31958	UGA-SAM1-266	INRA line SF_293	R	1	0	0	1	101.207	155	1	0
Ames 31959	UGA-SAM1-267	INRA line SF_009	M	0	0	0	1	101.207	155	0	1
Ames 31960	UGA-SAM1-268	INRA line SF_063	M	0	0	0	0		155	1	0
Ames 31961	UGA-SAM1-269	INRA line SF_210	M	0	0	0	0		155	0	1
Ames 31962	UGA-SAM1-270	INRA line SF_060	M	0	0	0	0		155	1	0
Ames 31963	UGA-SAM1-271	INRA line SF_296	R	1	1	0	0		170.155	0	1
Ames 31964	UGA-SAM1-272	INRA line SF_035	M	0	0	0	1	42,101,165	155	1	0
Ames 31965	UGA-SAM1-273	INRA line SF_076	M	0	0	0	1	42,101,165	155	1	0
Ames 31966	UGA-SAM1-274	INRA line SF_280	R	1	1	0	0		170	0	1
Ames 31967	UGA-SAM1-275	INRA line SF_169	M	0	0	0	1	42,101,165	155	1	0
Ames 31968	UGA-SAM1-276	INRA line SF_306	R	1	1	0	0		170.155	0	1
Ames 31969	UGA-SAM1-277	Saturn		0	0	0	1	42,101,165	155	1	0
Ames 31970	UGA-SAM1-278	INRA line SF_295	R	1	1	0	1	42,101,165	170	1	0
Ames 31971	UGA-SAM1-279	INRA line SF_307	R	1	0	0	0		155	1	0
Ames 31972	UGA-SAM1-280	INRA line SF_322	R	0	0	0	0		155	0	1
Ames 31973	UGA-SAM1-281	INRA line SF_320	R	1	0	0	0		155	1	0
Ames 31974	UGA-SAM1-282	INRA line SF_075	M	0	0	0	1	42,101,165	155	1	0
Ames 31975	UGA-SAM1-283	INRA line SF_292	R	1	1	0	1	101.207	170.155	0	1
Ames 31976	UGA-SAM1-284	INRA line SF_070	M	0	0	0	1	42,101,165	155	1	0
Ames 31977	UGA-SAM1-285	HOPI		0	0	0	0		155	1	0
Ames 31978	UGA-SAM1-286	INRA line SF_023	M	0	0	0	1	42,101,165	155	1	0
Ames 31979	UGA-SAM1-287	INRA line SF_279	R	1	1	1	1	101.207	170.155	0	1

Ames 31980	UGA-SAM1-288	INRA line SF_323	R	1	1	1	0		170	0	1
Ames 31981	UGA-SAM1-289	HA 250	M	0	0	0	1	42,101,165	155	1	0
CN29443	Saliut			0	0	0	1	42,101,165	155	1	0
CN30350	VNIIMK 6540			0	0	0	1	42,101,165	155	1	0
CN30351	VNIIMK 1646			0	0	0	1	42,101,165	155	1	0
CN30585	Zelanka 368			0	0	0	1	42,101,165	155	1	0
CN30592	Peredovik			0	0	0	1	42,101,165	155	1	0
CN30593	Smena			0	0	0	1	42,101,165	155	1	0
CN30603	Sputnik			0	0	0	1	42,101,165	155	1	0
CN32248	Luciole			1	1	1	1	42,101,165,207	170	1	1
CN32249	Clairsol			1	0	0	0		155	1	0
CN33280	Krasnodarets		M	0	0	0	1	42,101,165	155	1	0
CN33281	Armavirec			0	0	0	1	42,101,165	155	1	0
CN33284	Valley			0	0	0	1	42,101,165	155	1	0
CN33291	Saturn			0	0	0	1	42,101,165	155	1	0
CN33963	Cakinskij 269			0	0	0	1	42,101,165	155	1	0
CN34075	Corona			0	0	0	1	42,101,165	155	1	0
CN35798	Cakinskij 268			0	0	0	0		155	1	0
CN35799	Harkovskij 100			0	0	0	1	42,101,165	155	1	1
CN36154	CM338 X CM469DMR			1	1	0	1	42,101,165	170	1	1
CN36155	CM400 X CM469DMR			1	1	0	0		170	0	0
CN36537	CM28			0	0	0	0		155	1	0
CN36538	CM29			0	0	0	0		155	1	0
CN36540	CM31			0	0	0	0		155	1	0
CN36553	CM50			0	0	0	0		155	1	0
CN36555	CM53			0	0	0	0		155	1	0
CN36560	CM59			0	0	0	0		155	1	0
CN36562	CM63		M	0	0	0	0		155	1	0
CN36580	CM85			0	0	0	1	42,101,165	155	1	0
CN36581	CM86			0	0	0	1	42,101,165	155	1	0
CN36591	CM101			0	0	0	1	42,101,165	155	1	0

CN36595	CM105			0	0	0	1	42,101,165	155	1	0
CN36599	CM112			0	0	0	0		155	1	0
CN36601	CM119			0	0	0	1	42,101,165	155	1	0
CN36603	CM122			0	0	0	1	42,101,165	155	1	0
CN36605	CM126			0	0	0	0		155	1	0
CN36613	CM140			0	0	0	1	42,101,165	155	1	0
CN36628	CM188			0	0	0	0		155	1	0
CN36630	CM196			0	0	0	0		155	1	0
CN36642	CM227			0	0	0	0		155	1	0
CN36647	CM248			0	0	0	0		155	1	0
CN36648	CM251			0	0	0	1	42,101,165	155	1	0
CN36654	CM259		M	0	0	0	0		155	1	0
CN36665	CM275		M	0	0	0	1	42,101,165	155	1	0
CN36671	CM291			0	0	0	1	42,101,165	155	1	0
CN36674	CM302			0	0	0	0		155	1	0
CN36681	CM348			0	0	0	1	42,101,165	155	1	0
CN36683	CM352			0	0	0	1	42,101,165	155	1	0
CN36685	CM374			0	0	0	1	42,101,165	155	1	0
CN36696	CM561			0	0	0	0		155	1	0
CN36699	CM565			0	0	0	1	42,101,165	155	1	0
CN36702	CM568			0	0	0	0		155	1	0
CN36715	HA290			0	0	0	1	42,101,165	155	1	0
CN36716	HA301			0	0	0	1	42,101,165	155	1	0
CN36717	HA302			0	0	0	1	42,101,165	155	1	0
CN36721	CMS CM338			1	0	0	1	42,101,165	155	1	0
CN36730	CMS HA300			1	0	0	1	42,101,165	155	1	0
CN37365	Armavirsky 14			0	0	0	1	42,101,165	155	1	0
CN37369	CM526		M	0	0	0	1	42,101,165	155	1	0
CN37442	CMS NS-49			1	0	0	0		155	1	0
CN37444	CMS NS-71			1	0	0	1	42,101,165	155	1	0
CN40468	Elia			1	1	0	1	101.207	170	0	1

CN42251	CMS 2 CM 28			0	0	0	0		155	1	0
CN42254	Zelenka 368			0	0	0	1	42,101,165	155	1	0
CN42263	Sofia			0	0	0	1	42,101,165	155	1	0
CN42276	Hybrid 100, F2			1	1	0	0		170	0	1
CN42277	Hybrid 150			1	1	0	0		170	0	1
CN42283	Armavirsky 3497			0	0	0	1	42,101,165	155	1	0
CN45050	CM611			1	1	1	0		170	0	1
CN107886	AC Aurora			0	0	0	1	42,101,165	155	1	0
CN107887	AC Sierra			0	0	0	1	42,101,165	155	1	0
CN29474				0	0	0	1	42,101,165	155	1	0
CN29665				0	0	0	1	42,101,165	155	1	0
CN30581				0	0	0	1	42,101,165	155	1	0
CN30582				0	0	0	0		155	1	0
CN30583				0	0	0	1	42,101,165	155	0	0
CN30587				0	0	0	1	42,101,165	155	1	0
CN30594				0	0	0	1	42,101,165	155	1	0
CN30602				0	0	0	0		155	1	0
CN31597				0	0	0	0		155	1	0
CN31766				0	0	0	1	101.207	155	1	0
CN31834				0	1	1	1	101.207	170.155	0	1
CN31851				0	0	0	1	101.207	155	1	0
CN31926				0	0	0	1	42,101,165	155	1	0
CN32246				1	0	0	1	42,101,165	155	1	0
CN32247				1	1	1	1	42,101,165	170	1	1
CN32250				0	0	0	1	101.207	155	1	0
CN32251				1	0	0	1	42,101,165	155	1	0
CN32252				0	0	0	1	101.207	155	1	0
CN32256				0	0	0	1	42,101,165	155	1	0
CN32626				0	0	0	1	42,101,165	155	1	0
CN32627				0	0	0	1	42,101,165	155	1	0
CN33282				0	0	0	1	42,101,165	155	1	0

CN33283				0	0	0	1	42,101,165	155	1	0
CN33285				0	0	0	1	42,101,165	155	1	0
CN33287				0	0	0	1	42,101,165	155	1	0
CN33288				0	0	0	1	101.207	155	1	0
CN33290				0	1	0	1	42,101,165,207	170.155	1	1
CN33291				0	0	0	1	42,101,165	155	1	0
CN35597				1	0	0	1	42,101,165	155	1	0
CN36144				1	1	1	1	42,101,165	170	1	1
CN36180				0	0	0	1	42,101,165	155	1	0
CN36181				0	0	0	1	42,101,165	155	1	0
CN36523				0	0	0	1	42,101,165	155	1	0
CN36529				0	0	0	0		155	1	0
CN36530				0	0	0	0		155	1	0
CN36539				0	0	0	1	42,101,165	155	1	0
CN36584				0	0	0	1	42,101,165	155	1	0
CN36588				0	0	0	0		155	1	0
CN36589				0	0	0	1	42,101,165	155	1	0
CN36592				0	0	0	1	42,101,165	155	1	0
CN36594				0	0	0	1	42,101,165	155	1	0
CN36597				0	1	0	1	42,101,165	155	1	0
CN36598				0	1	0	0		155	1	0
CN36600				0	0	0	1	42,101,165	155	1	0
CN36621				0	0	0	1	42,101,165	155	1	0
CN36719	54-3			0	0	0	1	42,101,165	155	1	0
CN36732				1	0	0	1	42,101,165	155	1	0
CN36733				1	0	0	1	101.207	170	0	1
CN36734				1	0	0	1	101.207	170	0	1
CN37367				0	0	0	1	42,101,165	155	1	0
CN37441				0	0	0	1	101.207	155	1	0
CN39262				0	0	0	1	42,101,165	155	1	0
CN39649				0	0	0	1	42,101,165	155	1	0

CN39656				1	1	0	1	101.207	170	0	1
CN39662				0	0	0	1	42,101,165	155	1	0
CN39664				0	0	0	1	42,101,165	155	1	0
CN39665				0	0	0	1	42,101,165	155	1	0
CN42228				0	0	0	1	42,101,165	155	1	0
CN42229				0	0	0	1	42,101,165	155	1	0
CN42230				0	0	0	1	42,101,165	155	1	0
CN42231				0	0	0	1	42,101,165	155	1	0
CN42236				0	0	0	1	42,101,165	155	1	0
CN42243				0	1	0	1	101,207	170	0	1
CN42244				0	0	0	1	42,101,165	155	1	0
CN42252				1	0	0	1	101.207	155	1	0
CN42255				0	0	0	1	42,101,165	155	1	0
CN42267				1	1	1	0		170	0	1
CN42273				0	0	0	1	42,101,165	155	1	0
CN42275				0	0	0	1	42,101,165	155	1	0
CN42282				0	0	0	1	42,101,165	155	1	0
CN42285				0	0	0	1	42,101,165	155	1	0
CN42290				0	0	0	1	42,101,165	155	1	0
CN42293				0	0	0	1	42,101,165	155	1	0
CN42296				0	0	0	0		155	1	0
CN42297				0	0	0	1	42,101,165	155	1	0
CN42299				0	0	0	1	42,101,165	155	1	0
CN42325				0	0	0	1	42,101,165	155	1	0
CN42326				0	0	0	1	42,101,165	155	1	0
CN42329				1	0	0	1	42,101,165,207	155	1	0
CN42766				0	0	0	0		155	1	0
CN43612				1	1	1	0		170	0	1
CN43751				0	0	0	1	42,101,165	155	1	0
CN43752				0	0	0	1	101.207	155	1	0
CN45043				0	0	0	1	42,101,165	155	1	0

CN114388				0	0	0	0		155	1	0
CN114389				0	0	0	0		155	1	0
Referenz M		HA342		0	0	0	1	42,101,165	155	1	0

Supplementary Table 4: Overgo primers used in colony hybridization against two BAC libraries

Marker		Overgo primer name	Sequence 5'-3'
RAPD	OP-K13-454A	OP-K13_Ova	CCCTTCCATTAAAACCCAGCATCA
		OP-K13_Ovb	GTGTACATGTCGAAAGTGATGCTG
	OP-Y10-740A	OP-Y10_Ova	CGTGGGAGAGAGGTGGCCAACCCC
		OP-Y10_Ovb	AAAATGTCTCCTAATTCCCCAACC
	OP-H13-337A	OP-H13_Ova	TCTGTGTTCAATTGAGAATTCGCA
		OP-H13_Ovb	CCAATTTCATGGGTTGACGCTTAA
AFLP	E41M48_113A	E41M48_Ova	GTTCCACTTTCTGTCAACGAGGAA
		E41M48_Ovb	CAGCTACGAAAGCTAGTTCCTCGT
	E44M70_275A	E44M70_Ova	GGATTCATCCCTAGAAGTGCAAGAT
		E44M70_Ovb	CTTCCTTGAACACCTTCAATCTTGCA
	E62M52_249A	E62M52_Ova	GTGGGGTATGGAAAAAACAACTTGA
		E62M52_Ovb	GTGTCGTCTTCCTTGCATTCAAGTTG
BAC-end	67N04_B_end	67N04_B_end _Ova	ACGATGCCTTTTTCTTCTTTGAGG
		67N04_B_end _Ovb	GTAACTGGAGATGAGCCCTCAAAG

Supplementary Table 5: SSR primer combinations used for analyses of population structure.

Name	LG	Primer sequences * Forward/reverse 5'-3'	Expected size in bp	Reference
ORS728	1	CTCCATAGCAACCACCTGAAA / CCAAACTCTGAATGATACTTGTGAC	350	[70]
ORS822	1	CAATGCCATCTGTCATCAGCTAC / AAACAAACCTTTGGACGAAACTC	160	[70]
c4289	2	CACGCCATATGAAAGCAACTCT / TCATGGATGTTCATCACCAGAC	301	[68]
E28P08	2	GGTGGGTGTTTGATTTTGTGTG / TCTCCTCTCTCCCTTTGTCTGC	128	[68]
c0306	3	ACTGTCAACACCTCCTTCGACT / GGCTTACACTTCTCTCCATCTCAA	324	[68]
E11N15	3	GTTTCGCAGCTCTAGCATTGA / CCCCCTACAAGGCAACAAAATA	298	[68]
c1628	4	GCTGAAACGGGTCAATAAAGTC / TGAAGAAATGCTTCCAATCTGA	369	[68]
ORS785	4	CAAAATACCCAGGTCAAAGCA / CCTAGCTTATGGGACGTATGGA	180	[70]
c2443	5	GAAGTTGGGAGGGTTGTTCAAG / CCTCCTGTTGGAACACCAAAT	247	[68]
ORS547	5	TTGTCTTCATCTGCGTGTGA / TTGCTGTTGTTGATCGGTGT	180	[70]
c3464	6	TGTGCAGCGACGACTATAAAGA / CGTCAAACACAAATACTCCAACAA	250	[68]
ORS1256	6	GATGTTGATGTTGGTGAAGTTGC / CTCCGTCACCTTAAGCACTTGTA	210	[70]
c3258	7	AATTAGAAGGCTATGGCACAACA / CCTTATGGCCACCACATTACAT	198	[68]
c3797	7	CATTGAGGACGAGAAGCCAGT / GTTCCGTACCCTGTTTGAGCTT	331	[68]
c3240	8	TTTCGTGACCGAGAAAGGTAT / TGTGGCATACATAGAAATGATCTAA	273	[68]
ORS1161	8	CAACTACGTCACGATACTCGCC / GGAGCTGAAGCTGAAGACAAATC	236	[70]
c2104	9	TGGTCCCAACACTACTGATAAGG / TCAAAGGGATTCGTGAAAGTAGTT	171	[68]
G16E04	9	CTCAGGTGAACGGGATCCTTAT / CGGATTCGATTTCTTGCTTAGA	383	[68]
c1185	10	GGGCTATGGTGCGAATGTAGG / GCCTGTCAAATGTCCTTGTTGAT	277	[68]
ORS878	10	TGCAAGGTATCCATATTCCACAA / TATACGCACCGGAAAGAAAGTC	214	[70]
c2293	11	AACCGTAAATGAAATCGGTGTG / GAGGGCAAAGTTGGGATACTCT	329	[68]
c5783	11	GCCATCACAACATCAAATGG / AAGCTTGCACGTCCATACAC	140	[68]
F7O09	12	TTATGGAATGGAGTGGGAGTTG / TTGTTGGTTGATGGGATCTTGT	289	[68]
ORS778	12	CAACCAATCAATCCCACAAA / TGTTCACGCTTACACACATAATTG	384	[70]
c1731	13	GAAGTCTGCTTTGGTGTTGGTC / ATGGCTCTCTCATTTCCACTTG	377	[68]
ORS1030	13	TGATGTAGTTAAGGAAGTTGTG / CGATCAATTTATATGACCGAATTACC	430	[70]
B14H20	14	GACCGAATAGGTTTCAACGATAA / TTGCCTCTGCTCTCCTCTTTC	315	[68]
c0737	14	CAACTACCCATCACTGGCAAAT / ATCACCTCCAAACATCACAAGG	274	[68]

A11G17	15	TGAACTCTTGTGTTGGCATCT / AAATGTGGATTTATGTATCTCAGTAA	312	[68]
c2518	15	AAGCAGGTTGCATGAAGAGAAG / GTCGAAACGGGTCAGGTTGTAT	257	[68]
c2070	16	GGGTAATGCAAAGTACTAAGATGTG / GCATCATCCAACAAACTAGAAGG	235	[68]
E10D18	16	TCAAGCAATCAGACACCACATC / TGAACACAACCAAGAAATCCAA	304	[68]
c1779	17	CATGTCCCGATCAAAGAGTTGT / CCATATCCTGGTTGTTGTGGAG	383	[68]
I8D12	17	CTGAGTTTCGTGTACCATTTCTATTG / ACACCAATCAGTGGGTTTCATC	125	[68]

Supplementary Table 6: Primer combinations of the newly developed SCAR and PAMSA markers as well as other primers used in the publication

**Name**	**Sequence 5'-3'**	**Ta °C**	**Product size**	**Marker for**
67N04_F1a	TGCAAGATAGGCGACTGAGGGCTCATCTCCAATTA	65	170 bp	restorer
67N04_F2b	TGAGGGCTCATCTCCAGCTG	65	155 bp	maintainer
67N04_R	GGCTGCCATTAGTGAAGGAG			common
PPR621.5 F1	CAGTAATCTCCACATGAACATTG	62	164 bp	maintainer
PPR621.5 F2	CAATAATCTCCACATGAACATTC	62	164 bp	restorer
PPR621.5 Rev	CCGGATTGTGTTCCGATTAG			common
HRG01_for	TATGCATAATTAGTTATACCC	60	426 bp	restorer
HRG01_rev	ACATAAGGATTATGTACGGG			
HRG02_for	AAACGTGGGAGAGAGGTGG	65	738 bp	restorer
HRG02_rev	AAACGTGGGCTGAAGAACTA			
H13 CAPSfor	GTGTTAGACAAACATCACATA	58	207 bp + 101 bp (*Hinf*I digest)	restorer
H13 CAPSrev	GAGAATTCGCAGTTGGGTAC		165 bp +101 bp + 42 bp (*Hinf*I digest)	maintainer
orfH522_for	TGCCTCAACTGGATAAATTCAC	60	522 bp	CMS PET1
orfH522_rev	ACCGTTCTCTCACGAGTTGAAG			
coxII_for	CGAGAAATAGATGCTCAGCCTG	60	764 bp	Intern control
coxII_rev	GATAATGCGCAGTGGAAAGG			
atp9_for	GGTGCAAAATCAATAGGGGCCG	65	474 bp	Intern control
atp9_rev	ACCGAATGAATGCGTCACAAGG			
